# Vesicular Location and Transport of S100A8 and S100A9 Proteins in Monocytoid Cells

**DOI:** 10.1371/journal.pone.0145217

**Published:** 2015-12-14

**Authors:** Paramita Chakraborty, Per Bjork, Eva Källberg, Anders Olsson, Matteo Riva, Matthias Mörgelin, David Liberg, Fredrik Ivars, Tomas Leanderson

**Affiliations:** 1 Immunology Group, Lund University, Lund, Sweden; 2 Active Biotech AB, Lund, Sweden; 3 Division of Infection Medicine, Lund University, Lund, Sweden; Hormel Institute, University of Minnesota, UNITED STATES

## Abstract

We show here, by using surface biotinylation, followed by Western blotting or surface plasmon resonance analysis, that very low levels of S100A8 and/or S100A9 can be detected on the surface of THP-1 cells or freshly isolated human monocytes. This was supported by immune-electron microscopy where we observed membrane-associated expression of the proteins restricted to small patches. By using confocal microscopy we could determine that S100A8 and S100A9 protein in THP-1 cells or freshly isolated human monocytes was mostly present in vesicular structures. This finding was confirmed using immune-electron microscopy. Subcellular fractionation and confocal microscopy showed that these vesicular structures are mainly early endosomes and endolysosomes. Our subsequent studies showed that accumulation of S100A8 and S100A9 in the endolysosomal compartment is associated with induction of their release from the cells. Furthermore, an inhibitor of lysosomal activity could modulate the release of S100A8 and S100A9 in the extracellular milieu. Our current results suggest that the S100A8 and S100A9 proteins are primarily associated with certain kinds of cytosolic vesicles and may be secreted via an endolysosomal pathway.

## Introduction

The S100 protein family consists of over twenty members, most of which have been associated with various medical conditions [1, 2]. We have a particular interest in the S100A9 protein, which has been described to exert multiple extracellular and intracellular functions [2, 3]. S100A9 is a rather abundant protein in plasma where it is mostly found in a heterodimeric complex with another S100 protein, S100A8 [1]. However, S100A9 can also form homodimers and it has been shown that this form of the protein can mediate distinct biological functions compared to the S100A8/A9 heterodimer [4]. In addition, both S100A9 and S100A8/S100A9 can form higher order multimers, such as tetramers or even larger oligomers. Although the biological function of these various forms is currently unclear [3, 5] they may well be important for understanding S100A9 mediated signaling.

The intracellular transport and secretion of S100A9 has been studied previously [1]. It was shown that a Ca^++^ dependent mechanism could translocate cytoplasmic S100A8 and S100A9 to the cytoskeleton and the plasma membrane [6]. Interestingly, the authors also observed that S100A8 preferentially translocated to the cytoskeleton while S100A9 showed preference for the plasma membrane. The secretion mechanism for S100A8 and S100A9 is still undefined. It has been shown that treatment of monocytoid cells with various cytokines can trigger secretion [7], and it has also been shown that the secretion mechanism is distinct from the classical ER/Golgi route [8].

S100A8 and S100A9 have been shown to be pro-inflammatory ligands that can signal via the receptors RAGE and TLR4 [4, 9]. An unresolved question is how this signaling is regulated. Given the levels of S100A8/S100A9 observed in the circulation of healthy individuals, and the extreme levels observed in some disease states [1, 10], it is unlikely that the circulating forms of the S100A8/S100A9 proteins are biologically active since this would lead to a massive, systematic pro-inflammatory reaction. In our hands, homodimers of human S100A9 are potent TLR4 ligands while S100A8 homodimers and S100A8/S100A9 heterodimers are less potent [4]. Hence, a potential regulatory step in a pro-inflammatory cascade could be the assembly of the different stoichiometric forms of these two proteins.

We have previously shown that the human S100A9 protein is very unstable in the absence of S100A8 but is stabilized in cells exposed to pro-inflammatory stimuli [11]. This could represent one mechanism that would promote the formation of S100A9 homo-dimers. However, the details how homodimeric S100A9 is formed, transported out of the cell and secreted are unknown.

In this study, we have explored these processes using various techniques to better understand the basis for the formation, location and secretion of biologically active S100A9 homo-dimers/multimers.

## Materials and Methods

### Cell culture

The human monocytic leukemia cell line THP-1 was grown in RPMI Medium 1640—GlutaMAX^™^-I culture medium (Gibco; Life Technologies), supplemented with 10% fetal bovine serum Gibco (Invitrogen Corp, USA), 1 mM sodium pyruvate, 10 mM HEPES, 100 U/ml of penicillin and 100 μg/ml of streptomycin (all these supplements are from Gibco; Life Technologies) at 37°C in 5% CO_2_.

### Preparation of human monocytes

Human mononuclear cells were prepared from buffy coat by Ficoll separation (Ficoll Paque^™^ PLUS, GE Healthcare, Sweden), and followed by negative selection of CD14^+^ or positive selection of CD11b^+^ monocytes (MACS monocyte isolation kit II, Miltenyi Biotech, USA). According to the ethics committee of Lund Malmö there is no need for informed consent when material from buffy coats are used since these have been anonymised before donation to research. The negative monocyte fraction, which were used for biotinylation of viable cells, rendered in 18% yield, with a >95% purity when estimated by FACS analysis. The CD14^+^ cells were surface biotinylated and the number of cells were determined before the cell pellets (400 × *g*) were frozen at -70°C.

### Surface biotinylation of cells

In order to discriminate proteins localized on the cell surface from those found on intracellular membrane compartments, intact cells (viability > 98%) were reacted with a membrane non-permeable biotin reagent [12, 13]. Briefly, cells were washed three times in ice-cold phosphate-buffered saline (PBS), pH 8.0, and then suspended at a concentration of ~ 25 × 10^6^ cells/mL in the same buffer supplemented with 10 μM Zn^++^. To this suspension, sulfo-NHS-LC-biotin reagent (Thermo Scientific) was added at a final concentration of ~ 2 mM and allowed to react on ice for 40 min to avoid active internalization of the reagent. Finally, cells were washed three times with PBS containing 100 mM glycine to block excess of biotin reagent. The cell pellet was snap frozen on dry ice and stored at -70°C.

### Preparation of surface biotinylated plasma membranes

The frozen cell pellet was dissolved in 20 mM Tris-HCl, 0.25 mM sucrose, 10 mM KCl and 1.5 mM MgCl_2_, pH 7.6, with an EDTA-free cocktail of protease inhibitors and homogenized with 40 strokes using a glass homogenizer. After centrifugation (10,000 × *g* for 10 min), the supernatant was saved and the pellet re-suspended, homogenized and centrifuged as above. The combined supernatants were centrifuged at 105,000 × *g* for 1 h and the resulting pellet lysed with 1.5% v/v Nonidet P-40 in 75 mM Tris-HCl, pH 8.0, in the presence of protease inhibitors. The dissolved membrane pellet was collected and stored at -70°C for SPR and Western Blot analysis.

### Isolation of biotinylated plasma membrane proteins

Magnetic streptavidin beads (Dynabeads MyOne Streptavidin T1, Invitrogen Dynal AS, Oslo, Norway), 150 μL, were washed once with PBS containing 0.01% Tween-20 (PBST) and re-suspended in 100 μL PBST. Biotinylated membrane proteins were then immobilized by incubation of 500 μL lysate with 50 μL beads for 30 min at RT under gentle rotation. A magnet was applied and the non-biotinylated fraction collected and stored at -70°C until further analyzed. Beads were washed 3 times in PBST, aspirated under a stream of nitrogen and captured biotinylated proteins released by incubation (10 min at 65°C) in 100 μL LDS sample buffer (Invitrogen) for Western blot analysis.

### Surface plasmon resonance (SPR)

Surface and intracellular localization of S100 proteins was studied using SPR analysis in a Biacore 3000^™^ system (GE Healthcare, Uppsala, Sweden). In a first series of experiments, biotinylated proteins in lysates from biotinylated cells were captured on a streptavidin (SA) sensor chip until steady state was reached. Then antibodies raised against human S100A8 (mAb 8-5C2 from BMA Biomedicals, Augst, Switzerland), S100A9 (mAb 43/8; produced at Active Biotech AB), S100A8/S100A9 (mAb 27E10; BMA Biomedicals, Augst, Switzerland), RAGE/Fc (MAB11451; R&D Systems Minneapolis, MN, USA) or TLR4 (MAB14781; R&D Systems, Minneapolis, MN, USA) were injected for 2–3 min in 10 mM Hepes, 0.15 M NaCl and 0.005% Surfactant P20 (HBS-P), pH 7.4, containing 1 mM Ca^++^ and 10 μM Zn^++^. Bound antibodies were removed with a 15 μL pulse of 10 mM glycine-HCl, pH 2.0. In a second set of analyses, the non-biotinylated lysate was injected over 8-5C2, 43/8 and 27E10 immobilized via primary amines on a CM5 chip at a density of ~ 3 kRU. Specificity was investigated by incubating the non-biotinylated lysates with relevant antibodies prior to injection over the 8-5C2, 43/8 and 27E10 surfaces. In order to study whether S100A8 (Giotto Biotech, FI, Italy), S100A9 (produced at Active Biotech AB) or S100A8/S100A9 (R&D Systems) can bind to solubilized receptors these proteins were injected over the SA captured surface biotinylated proteins from THP-1 and monocytoid cells. Furthermore, heparin (Sigma-Aldrich) was tested as an inhibitor of S100A9 binding to TLR4 and RAGE. Evaluation of binding data was made using GraphPad Prism 4.0 and BIAevalution 3.0 software.

### Immunoprecipitation (IP)

Monocyte plasma membrane fractions were immuneprecipitated (IP) with biotinylated anti-bodies, followed by isolation of precipitated material with magnetic beads (Dynabeads MyOne Streptavidin T1) and elution with LDS buffer.

### Immunofluorescence microscopy

TNFα (10 ng/ml) treated THP-1 cells were plated onto poly-L-lysine coated slides from Thermo Scientific (Braunschweig, Germany) and incubated at 37°C for 30 min. Next, the culture medium was removed and the slides washed once with PBS. For intracellular staining, cells were fixed with 4% paraformaldehyde for 15 min at room temperature (RT) prior to permeabilization with PBS containing Tween 20 (0.05%) for 15 min at RT. After permeabilization, slides were saturated with PBS containing 10% donkey serum and 1% BSA for 30 min at RT, followed by Fc blocking with Human BD Fc Block (BD Biosciences) according to the manufacturers protocol. Cells were next stained with anti-S100A9 (mAb 1H9; Santa Cruz Biotechnology, CA, USA), rabbit-anti-S100A8 (kindly provided by Prof. Nancy Hogg, UK) or anti-S100A8/S100A9 (mAb 27E10). In some experiments double labeling was performed and cells were then labeled with anti-S100A9 (1H9) antibody together with anti-cathepsin D (Santa Cruz Biotechnology, CA, USA), anti-Rab5 or anti-Rab7 antibodies (Cell Signaling Massachusetts, USA), followed by the appropriate fluorophore-conjugated (Alexa 647 or Alexa 555) secondary antibody (Molecular Probes). Finally cells were incubated with Hoechst dye to visualize the nuclei. Between all incubation steps, cells were washed three times for 5 min with PBS-Tween unless specified otherwise. Slides were then mounted in aqueous mounting solution using cover glass (Fisher Scientific). The slides were analyzed using a Zeiss LSM 700 Confocal Microscope (Jena, Germany).

### Sample preparation and transmission immunoelectron microscopy analysis

0.2 × 10^5^ THP-1 cells were collected and centrifuged at 1000 *g* × 5 min. The pellet was washed in PBS and centrifuged once more at 1000 *g* for 5 min. Thereafter, cell pellet was re-suspended in 2.5% glutaraldehyde in 0.15 M sodium cacodylate, pH 7.4 (EM-fix solution), and incubated at RT overnight. Cells were then prepared for immunostaining and transmission electron microscopy as recently described [14]. In short, ultrathin sections of THP-1 cells were subjected to antigen retrieval with metaperiodate and then incubated overnight at 4°C with primary antibodies (anti-S100A8—from Prof. Nancy Hogg; anti-S100A9–43/8 or 1H9) diluted 1:100 in PBS. Detection was performed with the appropriate secondary antibody conjugated with 5 nm (S100A8) or 10 nm (S100A9) colloidal gold (Electron Microscopy Sciences, Fort Washington, Pa., USA; titer 1:10–1:20). Specimens were examined in a Philips/FEI CM100 BioTWIN at 60 kV accelerating voltage. Images were recorded with a side-mounted Olympus Veleta camera with a resolution of 2048 × 2048 pixels (2K × 2K).

### Subcellular fractionation using step gradient centrifugation

THP-1 cells were gently homogenized in homogenization buffer (250 mM sucrose, 3 mM imidazole, pH 7.4, with an EDTA free protease inhibitor cocktail (Roche, Mannheim, Germany), and post-nuclear supernatants (PNSs) were prepared by centrifuging the cell homogenate at 2,000 *g* for 10 min at 4°C. Endosome-enriched fractions were prepared from PNSs by centrifugation on a discontinuous sucrose gradient, following a standard protocol with slight modifications [15–18]. Briefly, PNSs were adjusted to 40.6% sucrose using a stock solution of 62% sucrose and loaded at the bottom of centrifugation tubes. This fraction was sequentially overlaid with 35% (1.5 volume of diluted PNS) and 25% sucrose (equal volume of diluted PNS) and the tubes finally filled up with homogenization buffer. The gradients were centrifuged at 210,000 *g* for 1.5 hours at 4°C (SW41 Ti rotor, Beckman Instruments, CA, USA). After centrifugation, 25% sucrose/homogenization buffer interphase (F1) and 35/25% sucrose interphase (F2) were collected and dialyzed against a dialysis buffer (10 mM HEPES, 0.15 M NaCl, pH 7.4) at 4°C using PUR-A-Lyzer^™^ Midi 3500 dialysis kit (Sigma-Aldrich, USA) and finally analyzed by Western blotting.

### Isolation of cell lysosomal fraction by differential ultracentrifugation

THP-1 cells were grown for 48 h in presence or absence of TNFα and subcellular fractionation to enrich for endolysosomes was performed using a previously described protocol [19, 20]. Briefly, cells were harvested and gently homogenized in homogenization buffer (250 mM sucrose, 20 mM HEPES-KOH, pH 7.2, 1.5 mM MgCl_2_, 10 mM KCl) at 5 × 10^7^ cells/ml using a Dounce homogenizer. The crude nuclear fraction was pelleted by centrifugation for 10 min at 12,000 × *g*. To isolate the lysosomal fraction, the post nuclear supernatant (PNS) was diluted 10-fold in homogenization buffer and centrifuged at 50,000 × *g* for 20 min. The supernatant (S50; post-endolysosomal fraction) was collected and the pellet (P50) was washed once in homogenization buffer and solubilized in 1% Triton X-100 dissolved in homogenization buffer.

### Western Blot analysis

The biotinylated and non-biotinylated plasma membrane fractions were analyzed by SDS-PAGE and Western blot. The samples were dissolved in LDS buffer with 20% dithioerythritol heated at 80°C for 5 min, and 5 μg protein was loaded on a 4–12% Bis-Tris gel and resolved (MES buffer) under reducing conditions. Immunoblotting was performed with mAb 8-5C2 and 1C10 (Novus Biologicals, CO, USA) as primary antibodies for detection of S100A8 and S100A9, respectively. In some experiments, whole cell lysate from treated or untreated THP-1 cells were prepared using a cell lysis buffer containing 10 mM HEPES (pH 7.9), 10 mM KCl, 0.5 mM PMSF, 1 mM DTT, 1.5% NP 40 and EDTA free protease inhibitor cocktail (Roche, Mannheim, Germany). Protein concentrations were measured using the Bradford method (Bio-Rad Protein Assay Dye Reagent, Munich, Germany) and Western blot analysis was done by a standard protocol. Briefly, aliquots (corresponding to 30μg of protein) from whole cell lysate, PNSs, post endolysosomal fraction, lysosome enriched fractions (P50) and endosome enriched fractions (F1 and F2) were resolved on 4–20% polyacrylamide gel (Bio-Rad, Solna, Sweden) under reducing conditions. Proteins were subsequently electro-transferred onto PVDF membrane (Roche, Mannheim, Germany). Membranes were blocked with 5% nonfat dry milk in PBS-0.05% Tween for 2 h before incubating overnight at 4°C with the appropriate primary antibody diluted 1:5000 in PBS-Tween. The primary antibodies were as follows: Mouse anti-human S100A8 (C-10), S100A9 (1H9) and goat anti-human cathepsin D (all of them purchased from Santa Cruz Biotechnology, CA, USA); rabbit anti-human β-tubulin (Novus Biologicals Inc., CO, USA); rabbit anti-human Rab5 antibodies (Biolegend, CA, USA), goat anti-human Rab7 antibodies (Abcam, Cambridge, UK). Next day membranes were washed 3 times in PBS-Tween, followed by incubation for 1 h at RT with anti-goat and anti-rabbit (both from Molecular Probes) or anti-mouse (Dako, Denmark) HRP conjugated secondary antibodies (diluted 1:10000), washed 3 times in PBS-Tween and finally developed using Bio-Rad Clarity^™^ western ECL substrate and analyzed using a ChemiDoc^™^ MP Imaging System (Bio-Rad Laboratories Inc., USA).

### Quantitative Real-Time PCR

Total RNA from THP-1 cells was extracted using GeneJET^™^ Kit. GoScript^**™**^ Reverse Transcriptase kit was used to reverse transcribe RNA to cDNA. qRT-PCR experiments were performed in triplicate to detect and quantify S100A8 and S100A9 mRNA using a Platinium^®^ SYBR^®^ Green qPCR supermix-UDG kit in an iCycler MyIQ^**™**^ instrument (BioRad, CA, USA). The primers used are A9 sense 5'-TTGACAGAGTGCAAGACGATG -3'; antisense 5'-GCTTCACAGAGTATTGGTGGAAGG -3', A8 sense 5'-CCTGAAGAAATTGCTAGAGACCG -3' antisense 5'-CACGCCCATCTTTATCACCAGA -3' Relative S100A8 and S100A9 expression was determined using the formula 2^(Rt—Et)^, where Rt and Et are the threshold cycles for the reference gene (beta actin) and the target gene, respectively.

### LDH assay

Release of lactate dehydrogenase was assayed from the culture supernatants of THP-1 cells to determine Cytotoxicity by using Pierce LDH cytotoxicity assay kit (Thermoscientific, Rockford, USA) following manufacturer’s protocol.

### Detection of extracellular S100A8/S100A9 dimer by ELISA

THP-1 cells were cultured in 24 well plates (5 × 10^5^ cells/ml) in RPMI-medium either with 10 ng/ml TNFα or 10 ng/ml TNFα + 10 ng/ml IL-10. In some experiments, cells were treated with both TNFα (10 ng/ml) and methylamine or brefeldin A (both from Sigma-Aldrich, St. Louis, MO, USA). Supernatants were collected after 48 h and assayed in triplicate for the production of S100A8/S100A9 and S100A9 by using an ELISA kit (from BMA Biomedical, Switzerland) following the manufacturer’s protocol.

### Statistical analysis

Data are shown as mean ± S.D. The unpaired Student’s t-test was used to evaluate the significant differences between groups, accepting p<0.05 as a level of significance.

## Results

### S100A9 can be detected on the cell surface of monocytes

**Fig 1:**
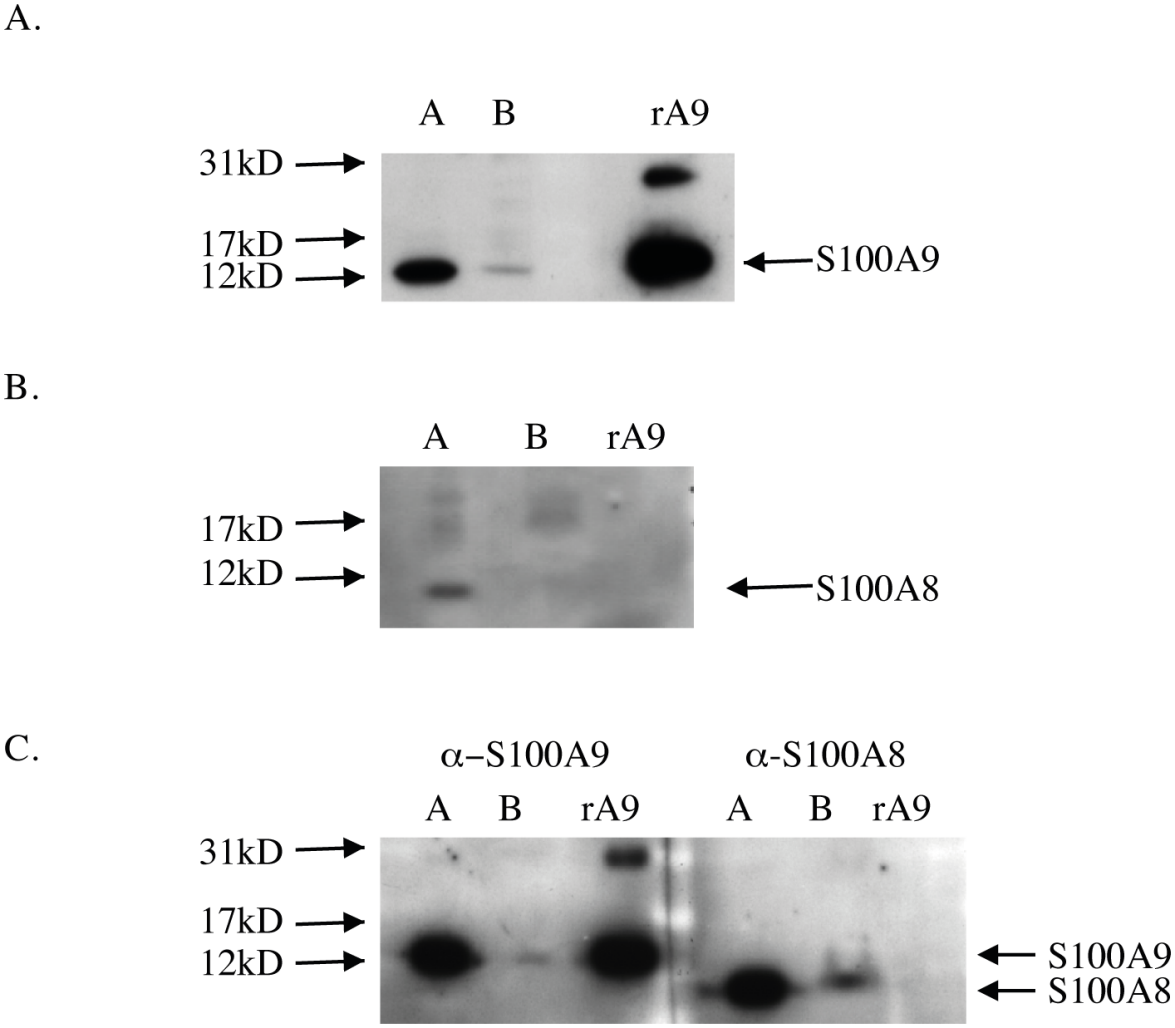
Western blot analysis under reducing conditions (10% SDS) of plasma membrane fractions prepared from cell surface biotinylated THP-1 cells and human monocytes. (A) S100A9 in the biotinylated plasma membrane fraction of THP-1 cells: non-biotinylated membrane fraction (lane a) and biotinylated cell surface fraction (lane b). (B) S100A8 expression in biotinylated THP-1 cells (same sample fractions as in (A). (C) Expression of S100A9 (left) and S100A8 (right) in the non-biotinylated membrane fraction (lane a) and biotinylated cell surface fraction (lane b) of human monocytes. Antibodies used were; α-S100A9 (NOVUS clone 1C10) and α-S100A8 (BMA; T1030; clone 8-5C2). 4 ng recombinant hS100A9 (rA9) was loaded.

We first wanted to investigate whether S100A8 and S100A9 would be exposed on the surface of monocytes. Surface biotinylation of cells makes it possible to discriminate between S100A9 present on the extracellular leaflet of the plasma membrane from that localized in membrane compartments inside the cell. We therefore performed surface biotinylation on THP-1 cells (as described in Materials and Methods) and separated the biotinylated and non-biotinylated fractions using magnetic beads. Western blot analysis of the non-biotinylated intracellular cell compartment (lane a, Fig 1A) and the biotinylated extracellular fraction (lane b, Fig 1A) revealed that the major fraction of S100A9 was located intracellularly, but some S100A9 could also be detected in the biotinylated fraction, indicating that S100A9 may be located at the cell surface of biotinylated THP-1 cells. The expression pattern of S100A8 was also determined (Fig 1B) but this protein could not be detected in the biotinylated membrane fraction (lane b, Fig 1B). The S100A8 and S100A9 expression pattern was also determined on purified intact CD14^+^ monocytes using the same experimental approach (Fig 1C). Although the S100A9 expression in the biotinylated plasma membrane fraction compared to the non-biotinylated membrane fraction of CD14^+^ monocytes seemed to be relatively similar to THP-1 cells, S100A8 was also detected in the biotinylated plasma membrane fraction of CD14^+^ monocytes (lane b, Fig 1C).

**Fig 2:**
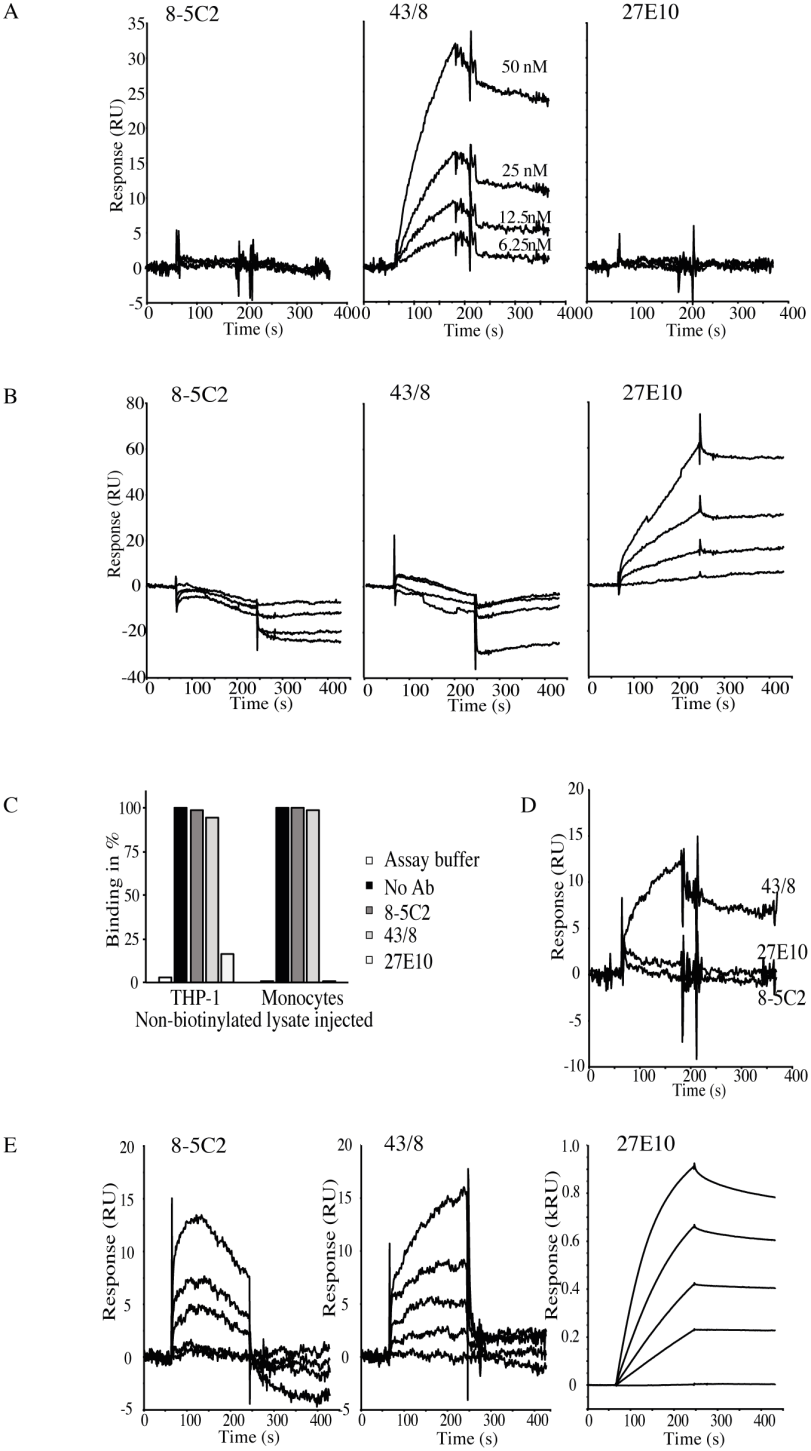
SPR analysis of S100A9 in isolated plasma membranes from THP-1 cells. (A) Binding of anti-S100A8 (8-5C2), S100A9 (43/8) and S100A8/S100A9 (27E10) to SA captured biotinylated plasma membranes. Antibodies were injected at 6.25–50 nM for 2 min at 30 μL/min in assay buffer (HBS-P with 1 mM Ca^++^ + 20 μM Zn^++^). A 15 μL pulse of 10 mM glycine-HCl was used for regeneration. Capture level ~ 2.1 kRU. Kinetic evaluation was made using a 1:1 model for mAb 43/8 at 25 and 50 nM. An affinity of 7.0 nM (*k*_on_ 1.9 × 10^5^ 1/Ms; *k*_off_ 1.3 × 10^−3^ 1/s) was calculated with maximal binding of 46 RU. (B) Injection of non-biotinylated THP-1 lysate over immobilized mAb 8-5C2, 43/8 and 27E10 (density ~ 3 kRU). Lysate, 0.45 mg/mL, diluted 5 to 40 times in assay buffer, injected for 3 min at 20 μL/min. Distinct binding was obtained when the THP-1 lysate was injected over the 27E10 surface whereas no binding was observed when lysate was passed over 8-5C2 and 43/8. (C) Binding of ten-fold diluted non-biotinylated THP-1 lysate to mAb 27E10 is inhibited after pre-incubation with 50 nM 27E10 but not with 8-5C2 or 43/8. Binding was expressed as % of response without antibody. (D) Sensorgrams obtained after injection of 50 nM mAb 8-5C2, 43/8 and 27E10 over SA captured (level ~ 1.1 kRU) solubilized membranes from surface biotinylated human monocytes. An affinity of ~ 6.1 nM ((*k*_on_ 2.7 × 10^5^ 1/Ms; *k*_off_ 1.6 × 10^−3^ 1/s) and maximal binding at ~ 12 RU was obtained after fit of sensorgram obtained with the 43/8 antibody to a 1:1 model. (E) Interaction of non-biotinylated lysate from human monocytes with 8-5C2, 43/8 and 27E10. Serially diluted lysate (5- to 40-fold in assay buffer) was injected over immobilized antibodies as in Fig 1B. Binding at high levels was demonstrated to mAb 27E10 (reaching steady state levels at ~ 1,000 RU) and was completely blocked when lysate (diluted 5-fold) was pre-incubated with 50 nM 27E10 but not with 8-5C2 or 43/8 (Fig 2C). Specific (i.e. displaceable by pre-incubation with anti-S100A9; data not shown) binding at much lower levels was also shown to 43/8 whereas non-specific binding was demonstrated to 8-5C2 (Fig 2E).

In order to study the presence of S100A8 and S100A9 on the cell surface in a native form, and also to increase the sensitivity of our analysis, we instead turned to surface plasmon resonance (SPR) technology. This technology also enables detection of native S100A8/A9 heterodimers recognized by the 27E10 antibody. Since high specificity was demonstrated for this antibody and for the 8-5C2 and 43/8 antibodies (S1 Fig), we could discriminate between S100A8, S100A9 and S100A8/S100A9 on the cell surface. Fig 2 shows such an analysis using surface-biotinylated plasma membranes isolated from THP-1 cells. In Fig 2A, the antibodies recognizing S100A8 and S100A9 as homo- and hetero-complexes were injected over biotin-labeled proteins captured on a SA chip. Dose-dependent binding was only observed for S100A9 (43/8 antibody) whereas neither S100A8 (8-5C2) nor the S100A8/S100A9 hetero-complex (27E10) was detected on the surface of THP-1 cells using SPR. Kinetic evaluation using a 1:1 model indicated an affinity of ~ 7 nM with maximal binding at 40–50 RU. In contrast, when the non-biotinylated lysate was injected over the same antibodies immobilized on a sensor chip, only the hetero-complex form of S100A8/S100A9 was detected (Fig 2B) and was displaced almost completely by pre-incubation of the lysate with 27E10 but not with 8-5C2 or 43/8 (Fig 2C).

When plasma membranes isolated from surface-biotinylated human monocytes were analyzed using the same experimental approach similar results were obtained. As is shown in Fig 2D, only the 43/8 epitope was recognized on the surface of these cells and with similar binding kinetics as observed for biotinylated THP-1 membranes (*K*_D_ ~ 6 nM). To confirm the the presence of S100A9 on the cell surface a control experiment was performed where human monocytes were pre-treated with Proteinase K prior to biotinylation. As shown in S2 Fig, pre-treatment with Proteinase K significantly reduced the S100A9 signal. This was also the case for known cell surface protein EMMPRIN, CD36 and RAGE. In the non-biotinylated lysate, the hetero-complex was by far the predominant form (Fig 2E) and binding was efficiently blocked only after pre-incubation of the non-biotinylated monocyte lysate with the 27E10 antibody (Fig 2C). Low but specific (i.e. displaceable with anti-S100A9) binding was also observed to the 43/8 antibody whereas non-specific binding was demonstrated to 8-5C2 (Fig 2E).

We also wanted to study whether TLR4 and RAGE could be detected on the cell surface after biotinylation with the plasma membrane non-permeable biotin reagent. As is shown in S3A Fig binding of both the anti-TLR4 and, to a lesser degree, the anti-RAGE antibody was demonstrated for biotinylated surface proteins from THP-1 cells. Both receptors could also be detected in biotin-labeled lysate from human monocytes but here the highest responses were obtained with the anti-RAGE antibody (S3B Fig). Having demonstrated the presence of both TLR4 and RAGE on the surface of monocytes and THP-1 cells, we injected S100A9 over the surface-biotinylated proteins from these cells. As is shown in S3C and S3D Fig. S100A9 demonstrated satiable binding to surface proteins from both cells with an affinity in the low nanomolar range (*K*_D_ ~ 2–3 nM) whereas negligible binding was observed for S100A8 and the S100A8/S100A9 hetero-complex. As S100A9 is known to possess a heparin-binding motif and heparin turned out to be a potent inhibitor of the interaction between S100A9 and TLR4 or RAGE, we also studied if heparin is able to block its binding to the biotinylated surface proteins. As is seen in S3E Fig heparin inhibited S100A9 binding to both cell lysates with an IC_50_ of 14 and 11 nM, respectively.

Taken together, these results suggest that S100A9 can be detected on the surface of human monocytes or THP-1 cells under native as well as denaturing conditions, whereas mostly the S100A8/S100A9 hetero-complex was detected in the intracellular compartment of the same cells.

### S100A9 is present in vesicular structures in monocytes

**Fig 3:**
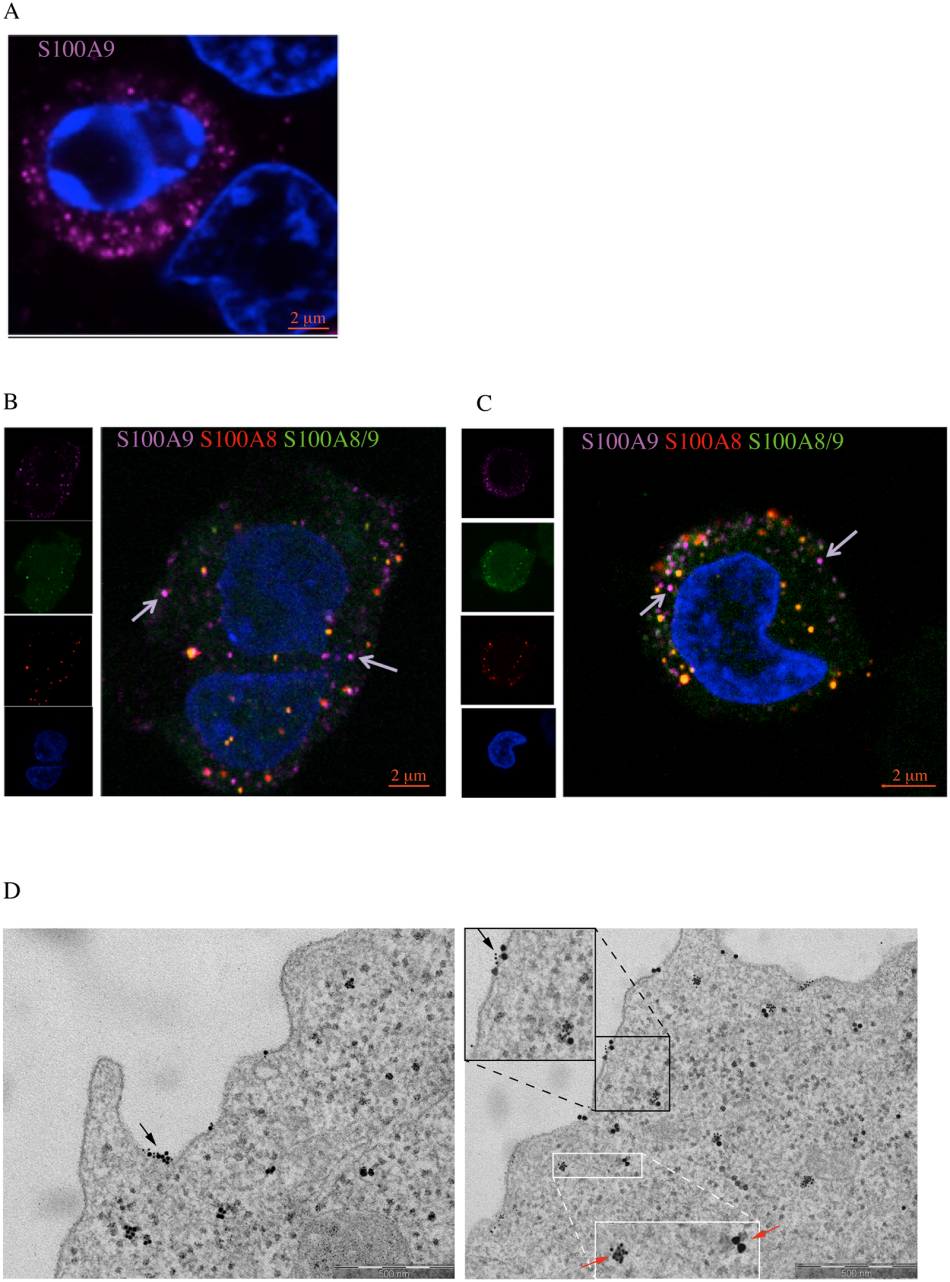
Distribution of hS100A8 and hS100A9 proteins in THP-1 cells by confocal and electron microscopy. THP1 cells were cultured for 48h in presence or absence of 10ng/ml TNFα and stained with antibodies against S100A9 (purple) (A). THP-1 cells were treated with either TNFα (B) or TNFα + IL10 (C); stained with anti-S100A8 (red), anti-S100A9 (purple) and anti-S100A8/S100A9 (green) antibodies. Arrows indicate the vesicles containing only hS100A9 or hS100A8. (D) Distribution of hS100A8 and hS100A9 proteins in THP-1 cells by Transmission Electron Microscopy. Black and white boxes represent magnification of areas where S100A8 and S100A9 hetero- or homodimers can be observed respectively. Further, black arrows highlight cytoplasmic vesicles or plasma membrane patches where hS100A9 (big dots) and hS100A8 (small dots) proteins co-localize, while red arrows point to vesicles containing only hS100A9 or hS100A8 homodimers.

We next wanted to investigate the subcellular distribution of S100A9 using confocal microscopy. We first stained stimulated THP-1 cells with antibodies against S100A8 and S100A9. We could detect different staining patterns using this approach. In some cells, S100A9 appeared to associate with the cytoskeleton (S4A Fig), while the more common staining pattern was a well-defined vesicular staining (Fig 3A). Moreover, in CD11b^+^ blood cells a similar variability was observed (S4B Fig). To further explore this variability we performed co-staining with phalloidin (actin) and anti-tubulin (S4A Fig). While a certain degree of co-staining was observed with anti-tubulin, no clear overlap was observed with phalloidin. In the next set of experiment we changed the experimental conditions in order to maximize the viability of the cells by performing all incubations in complete media (S4A Fig). Under these conditions no cytoskeletal staining pattern was detected. Hence, we conclude that in viable cells S100A9 protein is to a very large extent located to vesicular structures in monocytoid cells.

We then stained THP-1 cells with anti-S100A8, anti-S100A9 and anti-S100A8/S100A9 antibodies. As can be seen in Fig 3B, anti-S100A8 and anti-S100A8/S100A9 showed a very similar staining pattern and many vesicular structures stained with all three antibodies. We quantified the overlap using the Zen software from Zeiss, where the correlation coefficient between S100A8 and S100A9 was 0.68, while that between S100A8 and S100A8/S100A9 was 0.75. However, vesicular structures that only stained with anti-S100A9 antibodies were easily identified (arrows). Upon stimulation of THP-1 cells with TNFα together with IL10 a very similar staining pattern was observed (Fig 3C; correlation coefficient between S100A8 and S100A9 is 0.74 whereas between S100A8 and S100A8/S100A9 is 0.79).

To further clarify the intracellular distribution of S100A9 in monocytes we performed a Transmission Immuno Electron Microscopy analysis using ultrathin sections of THP-1 cells, which were fixed and analyzed using gold-labelled anti-S100A8 and anti-S100A9 antibodies. As shown in Fig 3D, S100A8 and S100A9 are co-localized in small vesicles distributed inside the cytoplasm. Also, vesicles that stained with only the anti-S100A9 or the anti-S100A8 antibody were easily detectable. Lastly, both S100A8 and S100A9 could be detected in focal patches associated with the plasma membrane of THP-1 cells using immune-electron microscopy. However, with this technology we cannot distinguish whether the protein is associated with the inner or the outer leaflet of the plasma membrane. Using surface biotinylation of intact cells only S100A9 was detected using SPR while S100A8/S100A9 heterodimers were associated with the membrane using TEM. An interpretation of these data could be that S100A9 can be associated with the outer leaflet of the plasma membrane but S100A8/S100A9 heterodimers are associated with the inner leaflet. We conclude from this analysis that S100A8 and S100A9 proteins are mostly associated with vesicular structures in monocytoid cells and that the composition of vesicles appears to be heterogeneous regarding S100A8 and S100A9 content. There might also be some selectivity with regard to the transport of the two proteins to the outer leaflet of the plasma membrane.

### Characterization of S100A8 and S100A9 containing vesicles

**Fig 4:**
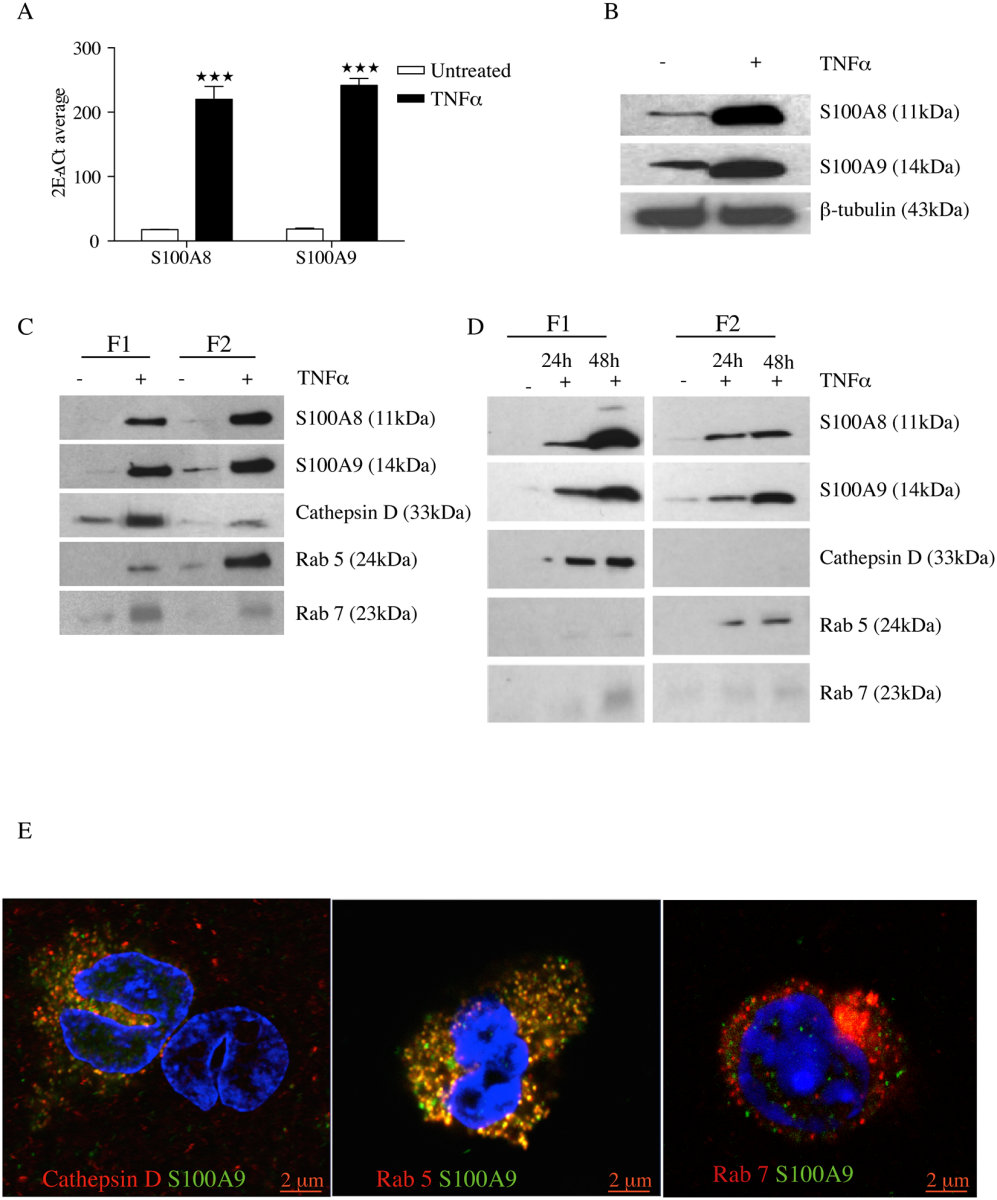
TNFα increases intracellular production of S100 proteins in THP-1 cells and S100A8 and S100A9 is co-localized in the vesicles along the endocytic pathway. THP-1 cells were cultured for 48h in presence or absence of 10 ng/ml TNFα. S100A8 and S100A9 mRNA expression was determined using qRT-PCR and was normalized to β-actin mRNA level. Data indicate mean ± SD of triplicate samples. Differences between treated and untreated groups are significant at ***P<0.001 by unpaired Student’s t test (A). Intracellular S100A8 and S100A9 protein expression was determined using Western blot (B). THP1 cells were treated with TNFα or left untreated. Cells were harvested either after 48 h (C) or after 24 h and 48 h (D); gently homogenized in homogenization buffer and post-nuclear supernatants (PNSs) were prepared. Using the PNSs as starting material, endosome enriched fractions were prepared using a discontinuous sucrose gradient centrifugation. Fractions were analysed by western blotting using the indicated antibodies. F1 is the interphase between 25% sucrose/homogenization buffer and F2 is the interphase between 35%/25% sucrose. (E) Immunofluorescence analysis of S100A9 localization in TNFα activated THP-1 cells; THP-1 cells cultured for 48 h with TNFα were fixed, permeabilized, stained with anti-S100A9 antibody (green) along with different vesicular marker antibodies anti-Rab5, anti-Rab7 and anti-cathepsin D (all of them marked in the red channel) and analyzed by confocal microscopy.

To get further insight in what type of vesicles that contained S100A8 and S100A9 we performed subcellular fractionation of THP-1 cells. To obtain fractions enriched for endocytic organelles we prepared post-nuclear supernatant from THP-1 cells followed by discontinuous sucrose gradient centrifugation as described in the Material and Methods section. We collected the different interphases and analyzed the distribution of specific markers for different endosomal organelles by Western blotting. To characterize the vesicles we used the markers Rab5 (early endosomes), Rab7 (late endosomes) and cathepsin D (endolysosomes) [21]. We performed the analysis both on naïve THP-1 cells and cells simulated with TNFα since we had observed that the level of S100A8 and S100A9 expression, both at the RNA (Fig 4A) and protein level (Fig 4B), increased significantly upon such stimulation with the highest expression observed after 48 hours of incubation.

Western blot analysis of the gradient fractions revealed that Rab7 and cathepsin D were enriched in the interface between 25% sucrose and homogenization buffer (F1), whereas the early endosomal marker Rab5 was enriched at the 35%/25% interphase (F2). Interestingly, S100A8 and S100A9 were found to be present in both interphases (Fig 4C). We next investigated the kinetics of S100 protein accumulation in different vesicle types. Thus, THP-1 cells were cultured in presence of TNFα, harvested at two different time points (24h and 48h) and analyzed as above. Western blot analysis showed that accumulation of both S100A8 and S100A9 in the F1 fraction increased over time while the vesicle markers did not, indicating an increased concentration of S100A8 and S100A9 in these vesicles over time. Interestingly, S100A9 showed a larger increase in the F2-fraction over time than S100A8, suggesting formation of S100A9 complexes with different stoichiometry in different vesicular compartments (Fig 4D).

To confirm the finding above, we co-stained TNFα treated THP-1 cells with anti-S100A9 antibody along with different vesicular marker antibodies (anti-Rab5, anti-Rab7 and anti-cathepsin D) and checked for co-localization with S100A9 using confocal microscopy. As shown in Fig 4E, co-localization of S100A9 with Rab5 and cathepsin D was detected (correlation coefficient between cathepsin D and S100A9 is 0.84 while that between RAB5 and S100A9 is 0.85) but we failed to detect co-localization of S100A9 with Rab7 (correlation coefficient 0.45). We also checked for the vesicle association of S100A9 and cathepsin D in human CD11b^+^ cells using Pearson analysis of 5 sections in 5 individual cells which gave a correlation coefficient of 0.53. As a comparison, the correlation coefficient between S100A9 and S100A8 positive vesicles was 0.82 (S5 Fig).

**Fig 5:**
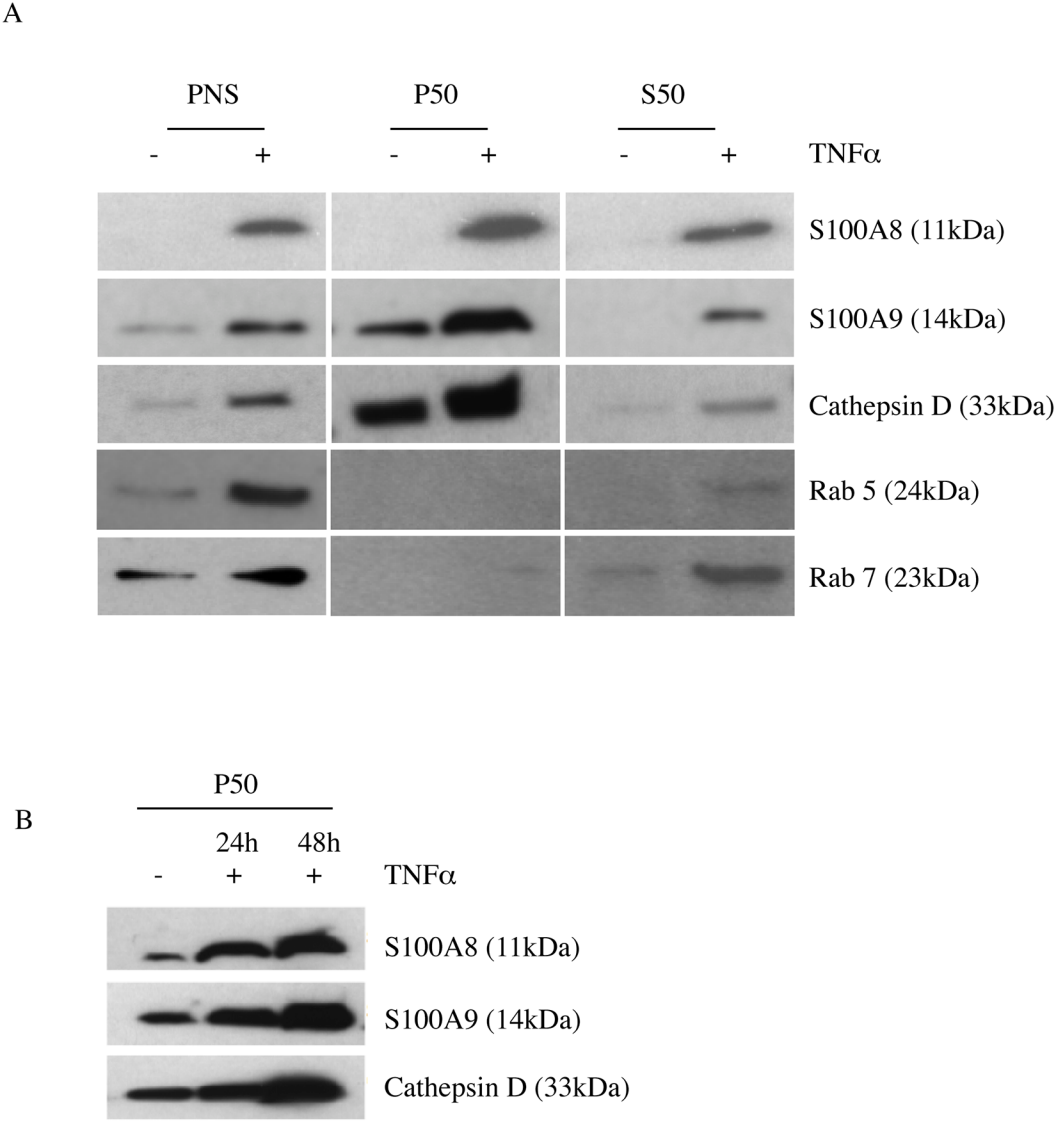
S100A8 and S100A9 are present in endo-lysosmal enriched fraction. THP-1 cells were grown for 48 h in presence or absence of TNFα. Cells were harvested after 48 h (A) or after 24h and 48h (B) and gently homogenized in homogenization buffer. Crude nuclear pellet was discarded by centrifugation. The post-nuclear supernatant (PNS) centrifuged at 50,000g for 20 min. The supernatant (S50; post endo-lysosomal fraction) was collected and pellet (P50) was solubilized in 1% Triton X-100 and aliquots from PNS, P50 fractions and S50 fractions were analyzed by Western blotting using the indicated antibodies.

Both the subcellular fractionation study and immunoflourescence analysis data showed that S100A8/S100A9 co-fractionated with the endolysosomal marker cathepsin D. Studies from several laboratories have shown that endolysosomes play an important role in secretion of various leaderless proteins through a non-classical pathway [19, 20, 22]. Therefore, next we wanted to know whether subcellular fractionation enriched for endolysosomes contained S100A8/S100A9. To this end, we enriched for the endolysosomal compartment by differential centrifugation following a standard protocol [19, 20] (see Materials and Methods section) both from naïve and TNFα-stimulated THP-1 cells and determined the presence of S100 proteins by Western blot. We found that both S100A8 and S100A9 were present in endolysosome-enriched fractions. Further, TNFα stimulation increased the accumulation of S100 proteins along with cathepsin D in the endolysosomal compartment (Fig 5A). We performed a kinetic study as well and the Western blot analysis in Fig 5B demonstrates that the amount of both S100A8 and S100A9, in parallel with cathepsin D, increased over time in the endolysosomal compartment.

### The increased S100A8 and S100A9 expression after TNFα stimulation results in protein secretion

**Fig 6:**
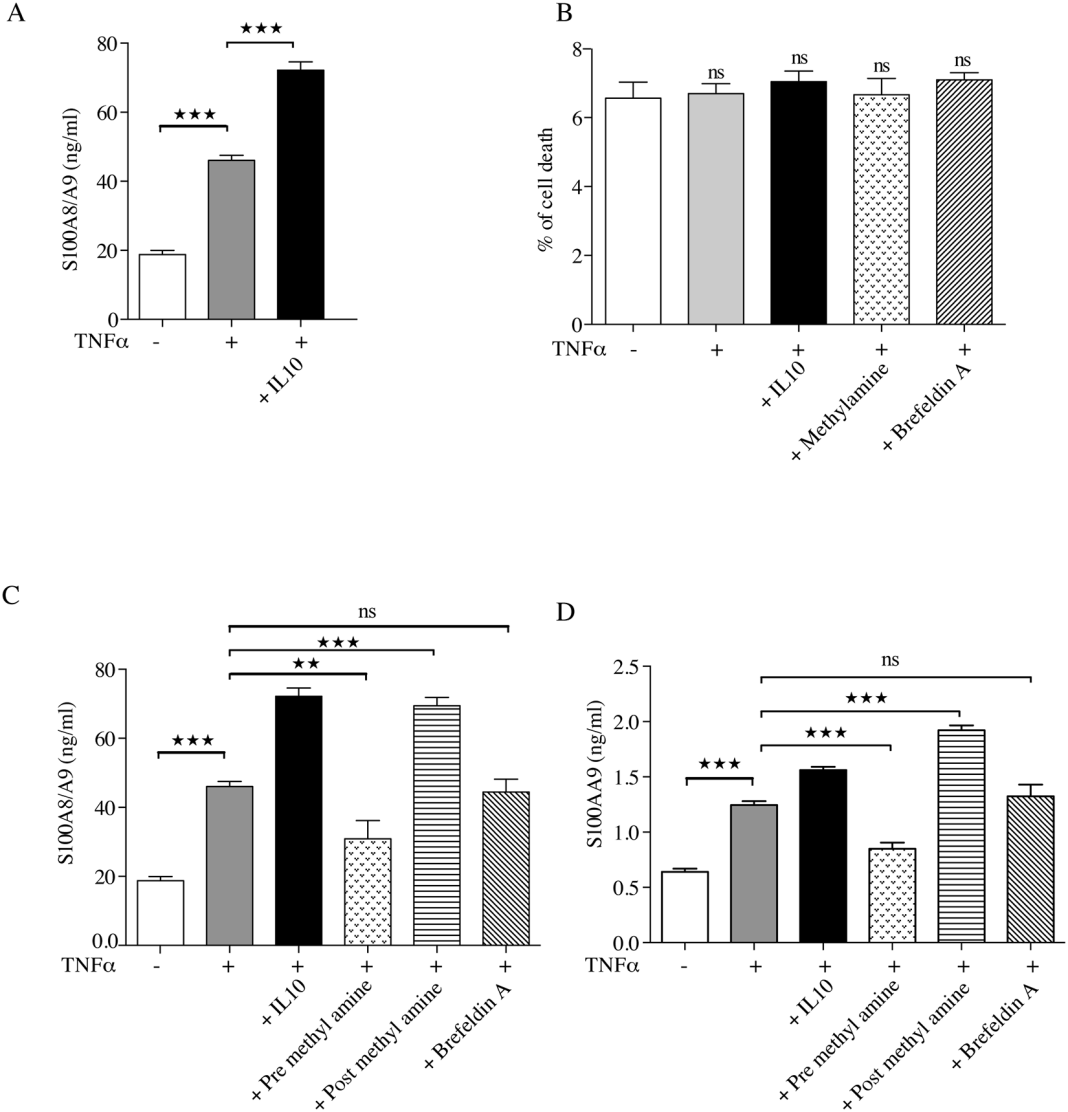
TNFα stimulates secretion of S100 proteins without causing cell death. THP-1 cells were cultured in triplicate either in the presence or absence of 10 ng/ml TNFα and TNFα + IL-10 (10 ng/ml). Culture supernatant was collected after 48h of incubation and S100A8/S100A9 dimer concentration was determined with ELISA following manufacturer’s protocol (A). Percent cell death was determined using LDH assay (B). In some experiments, THP-1 cells were stimulated with TNFα for 48 h in presence of methylamine (30 μM). In other experiments, cells were stimulated 12 h with TNFα without methylamine, followed by 36 h of culture with the drug. Alternatively, THP-1 cells were stimulated with TNFα; after 36 h, brefeldin A (5 μg/ml) was added and cells were cultured for another 12 h. Culture supernatant was collected after completion of incubation and S100A8/A9 heterodimer (C) and S100A9 (D) concentration was determined with ELISA following manufacturer’s protocol. Values are means of triplicates ± SD. Differences between various treatment groups are significant at **P<0.01, ***P<0.001 and ns = not significant by unpaired Student’s t test.

Lastly we wanted to investigate whether the increased expression of S100A8 and S100A9 and their subsequent accumulation in the endolysosomal compartment would lead to an increased secretion of those proteins. We therefore stimulated THP-1 cells for 48 hours with TNFα or TNFα together with IL10 and observed an increased level of S100A8/S100A9 in the supernatant after both treatments (Fig 6A). This increase did not correlate with increased cell death after stimulation, (Fig 6B), supporting that the S100A8/S100A9 protein in the supernatants was secreted rather than released by dying cells.

To get a link to the endolysosomal structures observed above we repeated this experiment in the presence of methylamine, a lysosomotropic drug that increases intra-lysosomal pH and thereby interferes with endolysosomal secretion [22]. We here observed that addition of methylamine at the initiation of cell culture decreased the secretion of S100A8/S100A9 while addition 12 hrs after initiation of culture rather increased the secretion from TNFα-treated cells (Fig 6C). However, brefeldin A, an inhibitor of ER/Golgi transport failed to block the release of these proteins, thereby confirming previous results [8]. Furthermore, analysis of S100A9 secretion provided similar results (Fig 6D), indicating that S100A8/S100A9 heterodimers and S100A9/S100A9 homodimers might be secreted via the same pathway. These findings are analogous to the results by Andrei et al [22] on IL1β secretion. Taken together, these data indicate a key role of the endolysosomal pathway in the secretion of S100A8/S100A9 from monocytoid THP-1 cells.

## Discussion

In this study we have found S100A8 and S100A9 to be localized in discrete clusters at the plasma membrane of THP-1 cells using EM. As a biochemical approach we performed cell surface biotinylation of both THP-1 cells and freshly isolated human monocytes followed by analysis of the biotinylated and non-biotinylated fractions using Western blotting and SPR. In the Western blot analysis the bulk of S100A8 and S100A9 protein was observed in the non-biotinylated, intracellular fraction but we could also observe S100A9, although with a weak signal, in the biotinylated fraction (Fig 1). Similar results were obtained using SPR analysis where S100A8 and S100A9 can be detected in their native state, thus enabling analysis of the 27E10 epitope found on the S100A8/S100A9 heterocomplex. The heterocomplex turned out to be the dominant form of S100A8 and S100A9 in the intracellular compartment of both cells (Fig 2B and 2E). However, in the fraction containing biotin-labeled surface proteins only S100A9 was detected (Fig 2A and 2D). Moreover, RAGE and TLR4 were found in this fraction (S3 Fig) and, when injected over these surfaces, S100A9 was bound with low nanomolar affinity in contrast to the negligible or low binding demonstrated for S100A8 or S100A8/S100A9. That S100A8/S100A9 heterodimers were not detected on the cell surface is in contrast to previous publications, where these could be readily detected using FACS staining [4, 23]. We think that this discrepancy is most likely explained by the more intense washing steps used in the surface biotinylation protocol, which might have removed soluble S100A8/S100A9 absorbed on the cell surface. The discrepancy between our Western blot findings in THP-1 cells versus human monocytes with regard to surface S100A8 expression might be due to the same phenomenon; i.e. that the human monocytes have absorbed S100A8/S100A9 complexes from the serum that was not completely removed in the washing steps. Taken together, these findings suggest that when S100A8 and S100A9 are exported from THP-1 and monocytic cells, S100A9 may interact with TLR4 and RAGE in an autocrine manner either directly as a homodimer or, when secreted as a heterocomplex, after release from S100A8 in the extracellular milieu.

The major finding of the current investigation is that S100A9 and S100A8 proteins are associated with cytoplasmic vesicles in monocytoid cells. Previous reports have shown that these proteins associate with certain neutrophil granules [24, 25]. The S100A8 and S100A9 proteins have previously been described as cytoplasmic proteins in human neutrophils [26] and monocytes [26, 27] and as associated with the cytoskeleton in monocytoid cells [6, 28]. Upon cellular activation S100A8/S100A9 was shown to associate with the cytoskeleton in neutrophils [28], monocytes [6, 7, 29] as well as in epithelial cell lines [30]. Further, in activated cells S100A8/S100A9 could be shown to translocate to the plasma membrane [24, 31] and also to associate with lipid rafts at this site [32].

The vesicles were studied both using confocal and electron microscopy (EM). Using both these techniques we observed that S100A8 and S100A9 could co-localize with cytoplasmic vesicles but we also observed occasional vesicles that co-localized with only one of the proteins (Fig 3B and 3C). However, vesicles containing S100A8 could only be observed using EM, while such vesicles were very rare, if not absent, when confocal analysis was performed (Fig 3D). The discrepancy between these data is most likely due to differences in sample preparation between the two techniques. These data do not allow us to firmly conclude whether the S100 proteins are located on the cytosolic leaflet of vesicular membranes or in the lumen of the vesicles. As discussed below, however, several pieces of evidence indicate that the proteins are actually secreted by some cells and therefore the proteins most likely reside in the lumen, at least in those vesicles associated with secretion of the proteins. This reasoning opens up the possibility that a mechanism behind the ability of monocytoid cells to secrete both S100A8/S100A9 heterodimers and S100A9 homodimers might involve a sorting mechanism into separate vesicles. The detailed mechanism for such a sorting process obviously merits further investigation.

Next, we attempted to characterize the type of vesicles that contained S100A8 and S100A9. Western blot analysis of the gradient fraction showed that both S100A8 and S100A9 are co-fractionating with Rab5, Rab7 and cathepsin D positive endocytic organelles (Fig 4C and 4D). Using confocal microscopy we observed co-localization of S100A9 and Rab5 and to some extent cathepsin D, markers for early endosomes and endolysosomes, but not with the late endosomal marker Rab7 (Fig 4E). This result is quite paradoxical, as late endosomes are derived from the vacuolar domains of early endosomes and late endosomes transiently fuse with each other to form larger bodies, and eventually fuse with lysosomes to give rise to endolysosomes. But lack of synchrony is characteristic of the endocytic pathway and organelles of this dynamic pathway undergo continuous maturation, transformation, fusion and fission. So, specific protein and lipid molecules are only partially useful as molecular markers for a particular organelle [33]. In any case, the question as to how S100 proteins are specifically sorted into these vesicular compartments among other cytosolic proteins remains to be answered. Also, it has already been reported that S100A8/A9 can modulate the function of other vesicle-associated proteins such as iNOS and NADPH oxidase [34–37]. Further studies are clearly needed to clarify the particular function of S100A8/S100A9 in these endocytic compartments.

TNFα stimulation of THP-1 cells upregulated the S100A8 and S100A9 expression both at the RNA and protein level (Fig 4A and 4B). This upregulation of protein expression was paralleled by secretion of the proteins out in the supernatant, an effect that was enhanced by the addition of IL10 (Fig 6A), as previously described in human monocytes [7]. S100 family proteins do not contain the signal sequences that would direct their secretion via the classical ER/Golgi route [2]. We could confirm the previous results of Rammes et al [8] since we observed that brefeldin A, an inhibitor of vesicular traffic through the ER and Golgi, did not inhibit TNFα-induced protein secretion. However, methylamine, a lysosomotropic drug increasing lysosomal pH could modulate secretion of S100A8/S100A9 from TNFα treated THP1 cells, which indicates an alternative pathway of secretion involving endolysosomes or secretory lysosomes (Fig 6C). This phenomenon resembles in some aspects the secretion of other leaderless proteins like IL1β, HMGB1 and HSP70, which also follow an alternative pathway of secretion [19, 20, 22]. For IL1β at least two other mechanisms of secretion has been documented which includes microvesicular shedding and exosome release [38, 39]. Moreover, IL-1β can be translocated into secretory lysosomes together with caspase 1 and then caspase 1 converts the premature IL-1β into its mature form [22]. Whether S100A8/A9 would acquire any such post-translational modifications during the transport through endolysosomes to resist degradation and remain stable in lysosomal compartment is unknown and merits further investigations. Several other alternative pathways for S100 proteins release have been proposed and may be operable under different circumstances. For example, it has been demonstrated recently that MDSC-derived exosomes contain S100A8/S100A9 [38] and human monocytes have previously been shown to release S100A8 and S100A9 via a novel pathway requiring an intact microtubule network and activation of protein kinase C [8]. Further, matrix vesicles released by macrophages associated with atherosclerotic lesions were also shown to contain S100A9 [40, 41]. That secretion mechanisms for a given protein may vary between cell types and stimuli has been previously described [22, 39].

Extracellular S100A8/S100A9 act as danger signal and amplify the inflammatory responses in infection, autoimmunity and cancer [1]. Furthermore, some reports indicate that S100A8/S100A9 released from inflamed tissue could enter systemic circulation and enhance susceptibility of distinct organ systems for autoimmunity and inflammatory disorders [42, 43]. Thus, a detailed knowledge of the release-mechanisms of S100A8/S100A9 and an understanding of the formation of different S100A8 and S100A9 complexes under different inflammatory conditions will aid in the understanding of the pathogenesis of autoimmune/inflammatory disorders and the developing of novel treatment modalities.

## Supporting Information

S1 Fig Test of S100A8, S100A9 and S100A8/A9 antibody specificity using SPR. Sensorgrams obtained after injection (3 min at 20 μL/min in HBS-P containing 1 mM Ca^++^ and 20 μM Zn^++^) of S100A8 (100 nM), S100A9 (12.5 nM) and S100A8/S100A9 (12.5 nM) over biotin-labeled 8-5C2, 43/8 and 27E10 captured on a SA chip (level in each flow cell: ~ 2.5 kRU). Specificity for the respective antigen was demonstrated. An eight-fold higher concentration of S100A8 was used since it reacted with 8-5C2 with a much lower response. Some binding of S100A8/S100A9 to 43/8 was observed which most probably is due to the presence of low amounts of S100A9 as the hetero-complex is obtained by association of S100A8 and S100A9 separately produced in *E*. *coli*.
(TIF)

S2 Fig Detection of S100A9 and integral receptor proteins on the surface of THP-1 cells before and after proteinase K treatment. Surface biotinylated plasma membrane proteins from THP-1 cells, with or without proteinase K treatment (1 mg/ml PBS for 40 min at 4°C) before biotinylation, were captured in separate flow cells on a SA chip. Sensorgrams obtained after injection (3 min at 20 μL/min) of 50 nM (A) anti-hS100A9 (mAb 43/8), (B) anti-hRAGE (AF1145), (C) anti-hCD36 (AF1955) or (D) anti-hEMMPRIN (AF972) after subtraction of the response in a SA reference cell. Upper curves represent untreated THP-1 cells and lower curves THP-1 cells treated with proteinase K.
(TIF)

S3 Fig Binding of S100A9 to TLR4 and RAGE detected in plasma membranes from THP-1 cells and monocytes. Sensorgrams obtained after injection (2 min at 20 μL/min) of 50 nM anti-human TLR4 (MAB14781) or anti-RAGE (MAB11451) over biotin-labeled surface proteins from THP-1 cells (A) or monocytes (B) captured on a SA chip at levels of 2.1 and 2.6 kRU. C and D: 25 nM S100A8, S100A9 and S100A8/S100A9 injected (3 min at 20 μL/min) over SA captured surface proteins from THP-1 cells (C; level 3.2 kRU) or human monocytes (D; level 2.3 kRU) in HBS-P buffer containing 1 mM Ca and 20 μM Zn. K_D_ values of 3.3 and 2.3 nM were calculated after fit of sensorgrams to a 1:1 model. E: Inhibition of S100A9 binding to THP-1 (black bars) or human monocyte (grey bars) surface proteins by 5 to 50 nM heparin. IC_50_ values of 14 and 11 nM were calculated. Regeneration was made with 10 mM glycine-HCl, pH 2.0, (A-B) or a 30 μL pulse of 3 mM EDTA in HBS-P buffer (C-D).
(TIF)

S4 Fig Cellular distrubution of hS100A9 proteins in THP-1 cells and monocytes by confocal microscopy. THP-1 cells were cultured for 48 h in presence of 10 ng/ml TNFα followed by attachment to polylysine-coated glass slides in fully supplemented RPMI or PBS for 90 min, thereafter fixed in ice-cold methanol and stained with anti-S100A9 (red), and cytoskeleton markers indicated in figure in green (A). Human PBMC were treated with TNFα followed by attachment to polylysine-coated glass slides in fully supplemented RPMI thereafter fixated in PFA and stained with anti-S100A9 (B) and Phalloidin in green.
(TIF)

S5 Fig Vesicular association of hS100A8 and hS100A9 proteins in human monocytes by confocal microscopy. CD11b^+^ human monocytes were treated with 10 ng/ml TNFα for 48h and stained with anti-S100A9 (red), anti-S100A8 (green) and cathepsin D (green) antibodies. Pearson’s analysis was performed on five sections in five individual cells using SlideBook6 (3i) software and Otsu Automatic settings. The correlation coefficients were for S100A9/cathepsin D 0.53, for S100A9/S100A8 0.82 and for S100A9/Hoechst -0.9. Very similar results were obtained in an independent experiment.
(TIF)

S6 Fig Full blots of Fig 4B. (TIF)

S7 Fig Full blots of Fig 4C. (TIF)

S8 Fig Full blots of Fig 4D. (TIF)

S9 Fig Full blots of Fig 5A. (TIF)

S10 Fig Full blots of Fig 5A and 5B. (TIF)

S11 Fig S4 Fig PFA fixed. (TIF)
