# Supplementary figures and images for: Vesicular Location and Transport of S100A8 and S100A9 Proteins in Monocytoid Cells
Source: PLoS One. 2015 Dec 14;10(12):e0145217. doi: 10.1371/journal.pone.0145217 (PMC4678419; doi:10.1371/journal.pone.0145217)

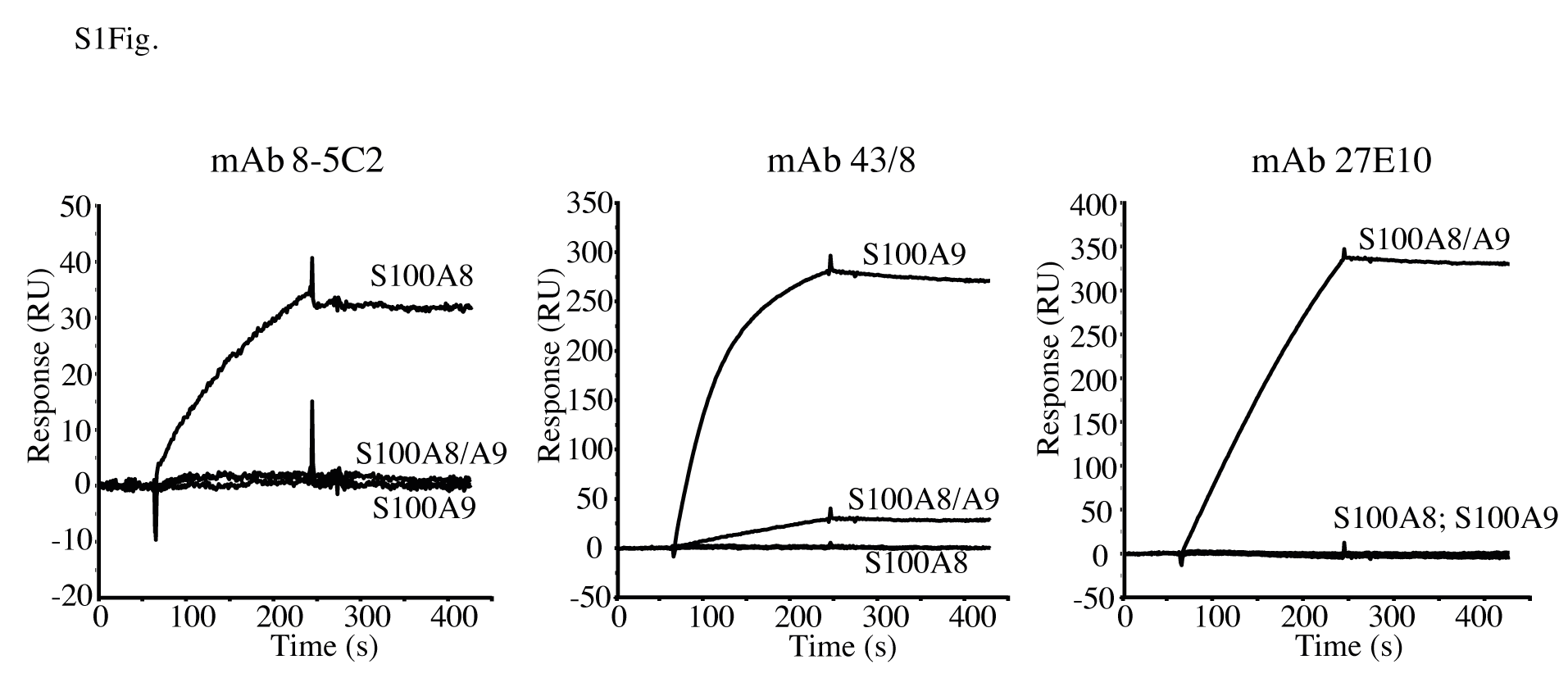

Supplement: S1 Fig — Sensorgrams obtained after injection (3 min at 20 μL/min in HBS-P containing 1 mM Ca++ and 20 μM Zn++) of S100A8 (100 nM), S100A9 (12.5 nM) and S100A8/S100A9 (12.5 nM) over biotin-labeled 8-5C2, 43/8 and 27E10 captured on a SA chip (level in each flow cell: ~ 2.5 kRU). Specificity for the respective antigen was demonstrated. An eight-fold higher concentration of S100A8 was used since it reacted with 8-5C2 with a much lower response. Some binding of S100A8/S100A9 to 43/8 was observed which most probably is due to the presence of low amounts of S100A9 as the hetero-complex is obtained by association of S100A8 and S100A9 separately produced in E. coli. (TIF) [file pone.0145217.s001.tif]

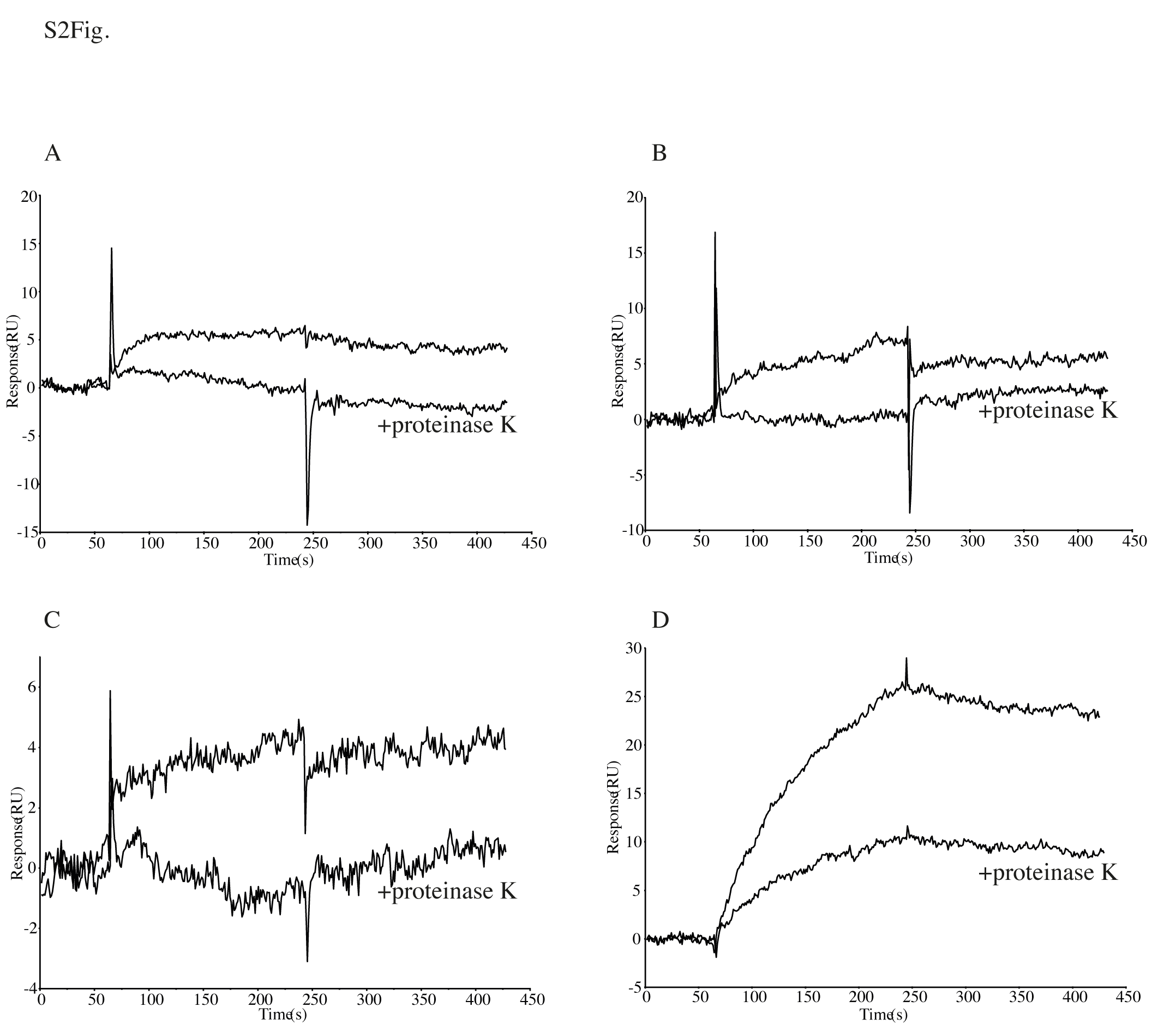

Supplement: S2 Fig — Surface biotinylated plasma membrane proteins from THP-1 cells, with or without proteinase K treatment (1 mg/ml PBS for 40 min at 4°C) before biotinylation, were captured in separate flow cells on a SA chip. Sensorgrams obtained after injection (3 min at 20 μL/min) of 50 nM (A) anti-hS100A9 (mAb 43/8), (B) anti-hRAGE (AF1145), (C) anti-hCD36 (AF1955) or (D) anti-hEMMPRIN (AF972) after subtraction of the response in a SA reference cell. Upper curves represent untreated THP-1 cells and lower curves THP-1 cells treated with proteinase K. (TIF) [file pone.0145217.s002.tif]

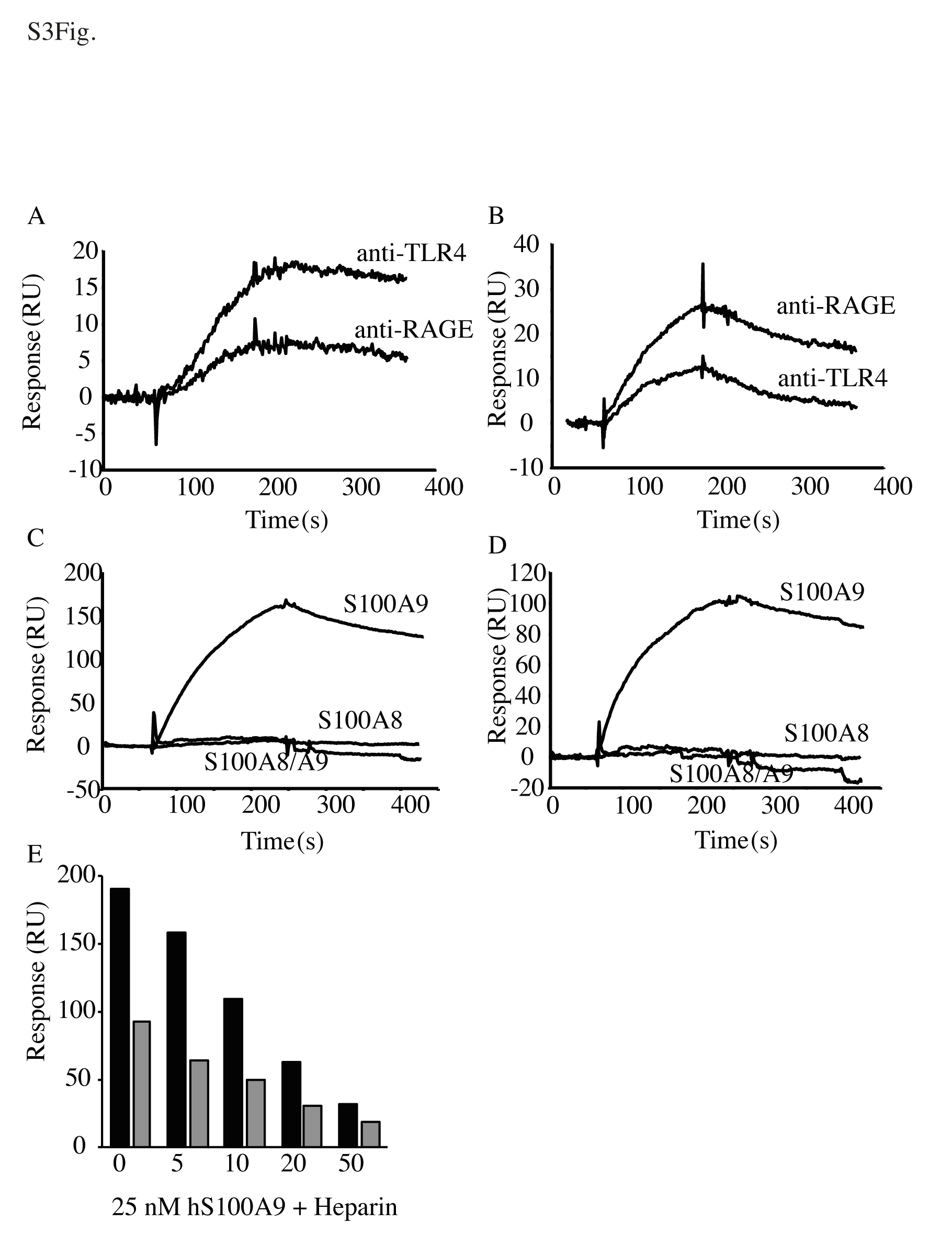

Supplement: S3 Fig — Sensorgrams obtained after injection (2 min at 20 μL/min) of 50 nM anti-human TLR4 (MAB14781) or anti-RAGE (MAB11451) over biotin-labeled surface proteins from THP-1 cells (A) or monocytes (B) captured on a SA chip at levels of 2.1 and 2.6 kRU. C and D: 25 nM S100A8, S100A9 and S100A8/S100A9 injected (3 min at 20 μL/min) over SA captured surface proteins from THP-1 cells (C; level 3.2 kRU) or human monocytes (D; level 2.3 kRU) in HBS-P buffer containing 1 mM Ca and 20 μM Zn. KD values of 3.3 and 2.3 nM were calculated after fit of sensorgrams to a 1:1 model. E: Inhibition of S100A9 binding to THP-1 (black bars) or human monocyte (grey bars) surface proteins by 5 to 50 nM heparin. IC50 values of 14 and 11 nM were calculated. Regeneration was made with 10 mM glycine-HCl, pH 2.0, (A-B) or a 30 μL pulse of 3 mM EDTA in HBS-P buffer (C-D). (TIF) [file pone.0145217.s003.tif]

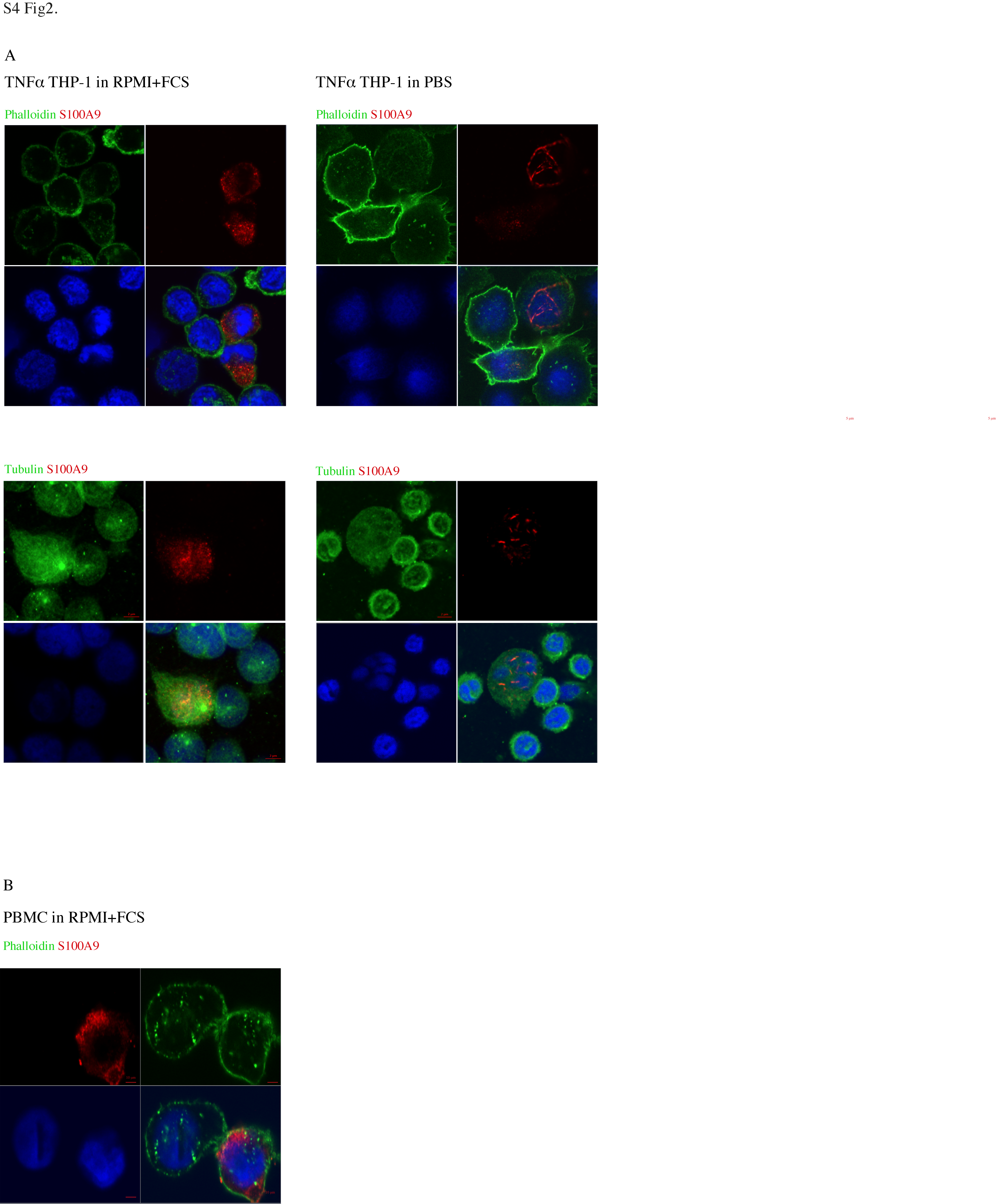

Supplement: S4 Fig — THP-1 cells were cultured for 48 h in presence of 10 ng/ml TNFα followed by attachment to polylysine-coated glass slides in fully supplemented RPMI or PBS for 90 min, thereafter fixed in ice-cold methanol and stained with anti-S100A9 (red), and cytoskeleton markers indicated in figure in green (A). Human PBMC were treated with TNFα followed by attachment to polylysine-coated glass slides in fully supplemented RPMI thereafter fixated in PFA and stained with anti-S100A9 (B) and Phalloidin in green. (TIF) [file pone.0145217.s004.tif]

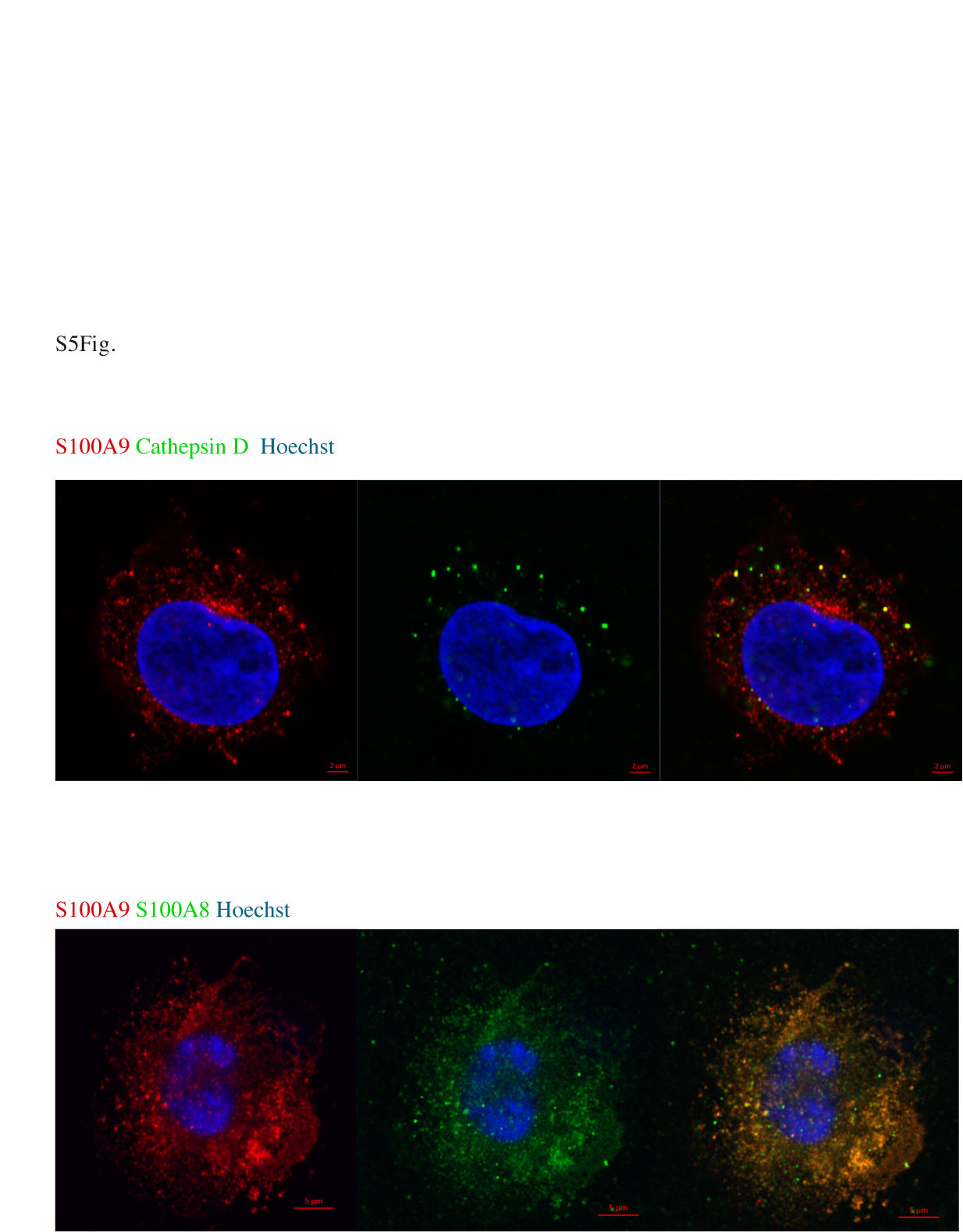

Supplement: S5 Fig — CD11b+ human monocytes were treated with 10 ng/ml TNFα for 48h and stained with anti-S100A9 (red), anti-S100A8 (green) and cathepsin D (green) antibodies. Pearson’s analysis was performed on five sections in five individual cells using SlideBook6 (3i) software and Otsu Automatic settings. The correlation coefficients were for S100A9/cathepsin D 0.53, for S100A9/S100A8 0.82 and for S100A9/Hoechst -0.9. Very similar results were obtained in an independent experiment. (TIF) [file pone.0145217.s005.tif]

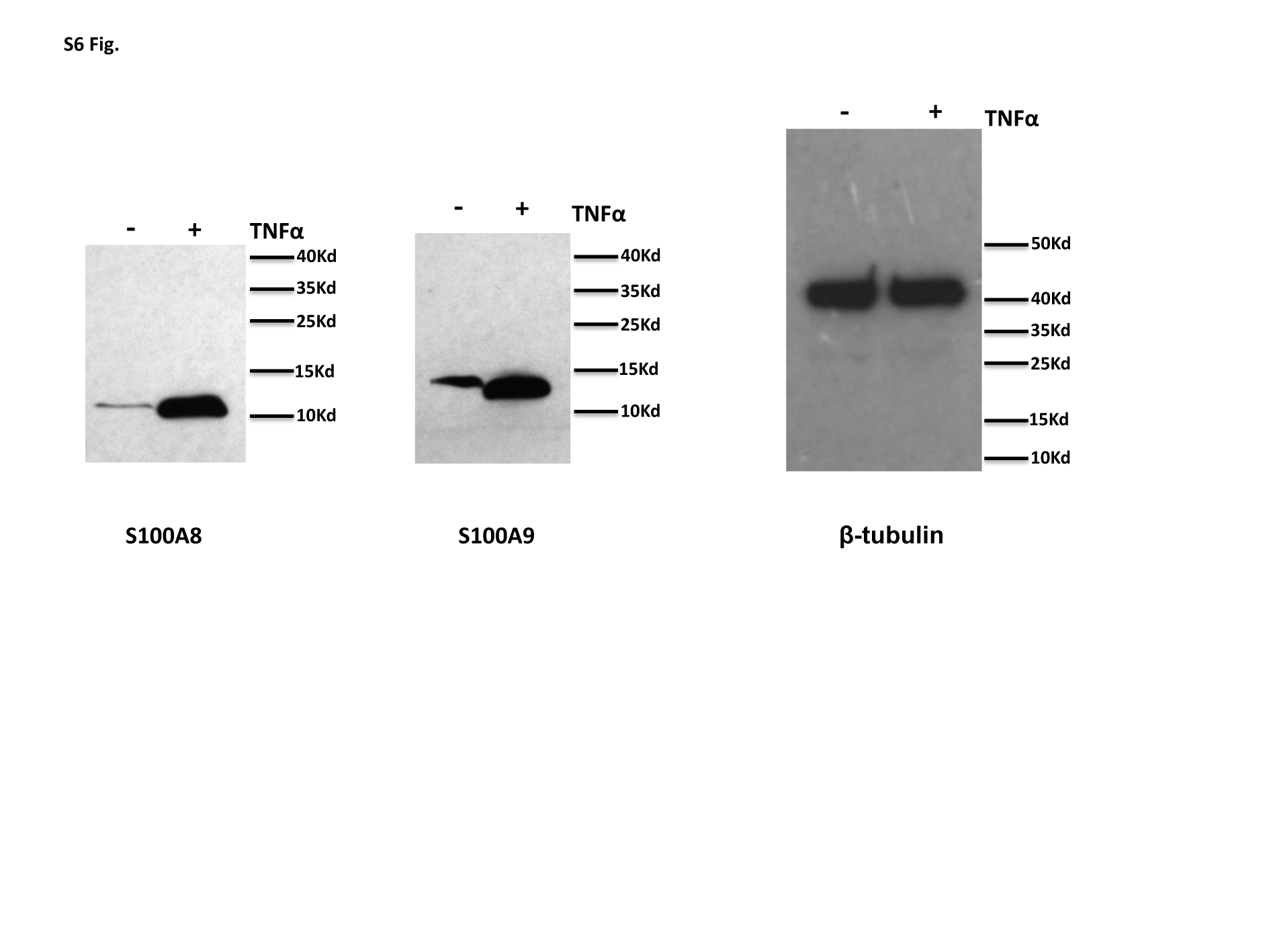

Supplement: S6 Fig — (TIF) [file pone.0145217.s006.tif]

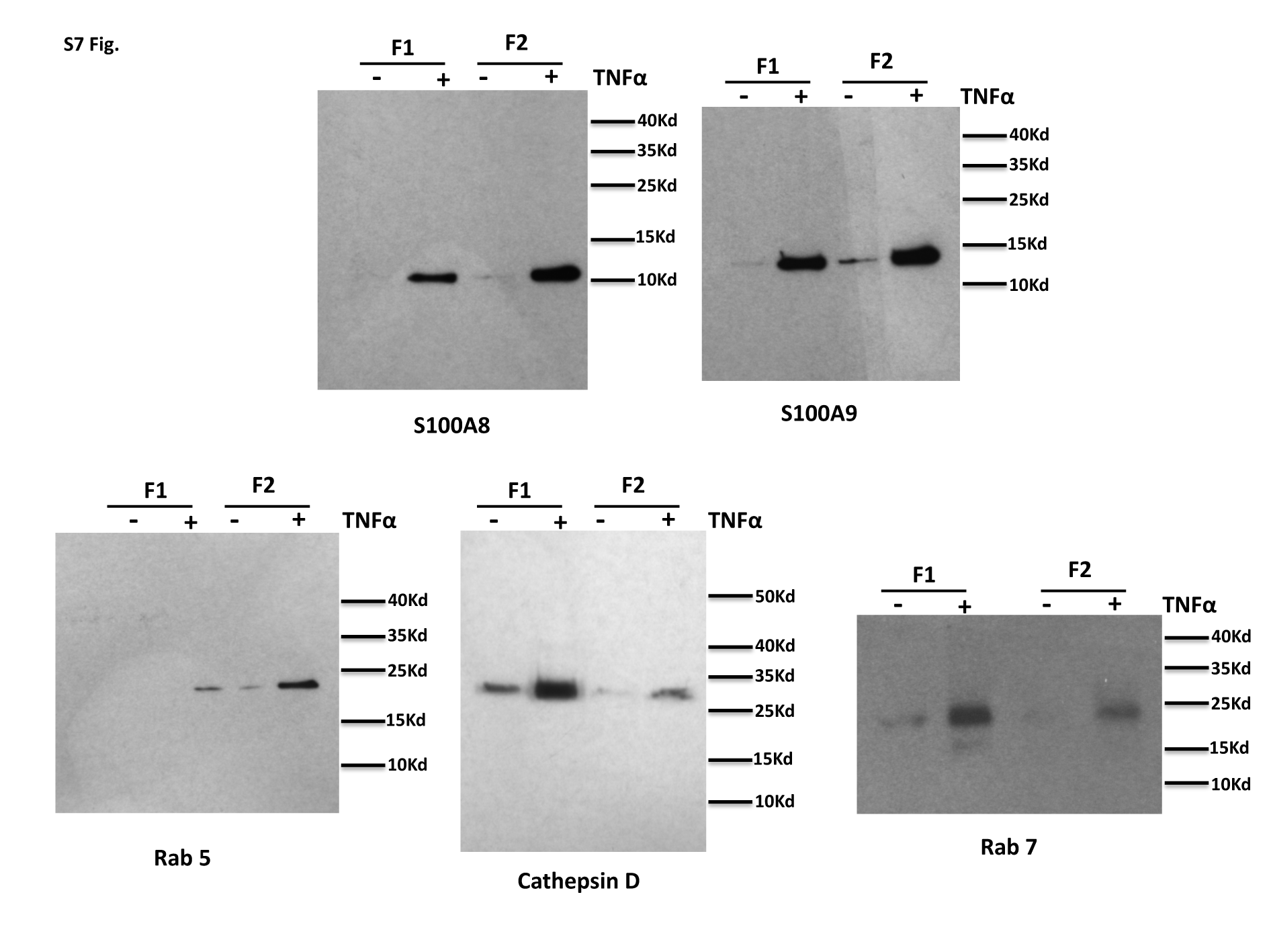

Supplement: S7 Fig — (TIF) [file pone.0145217.s007.tif]

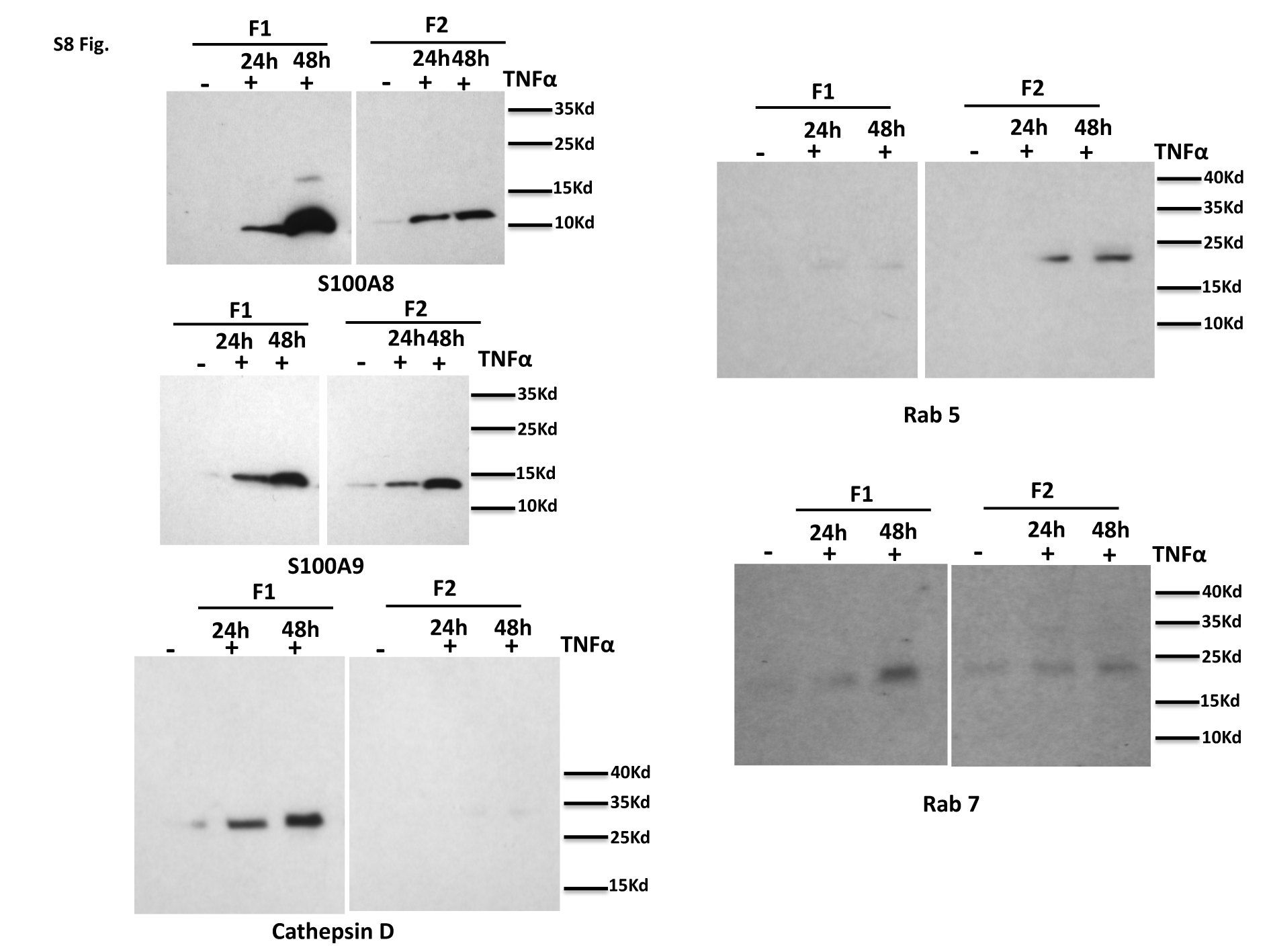

Supplement: S8 Fig — (TIF) [file pone.0145217.s008.tif]

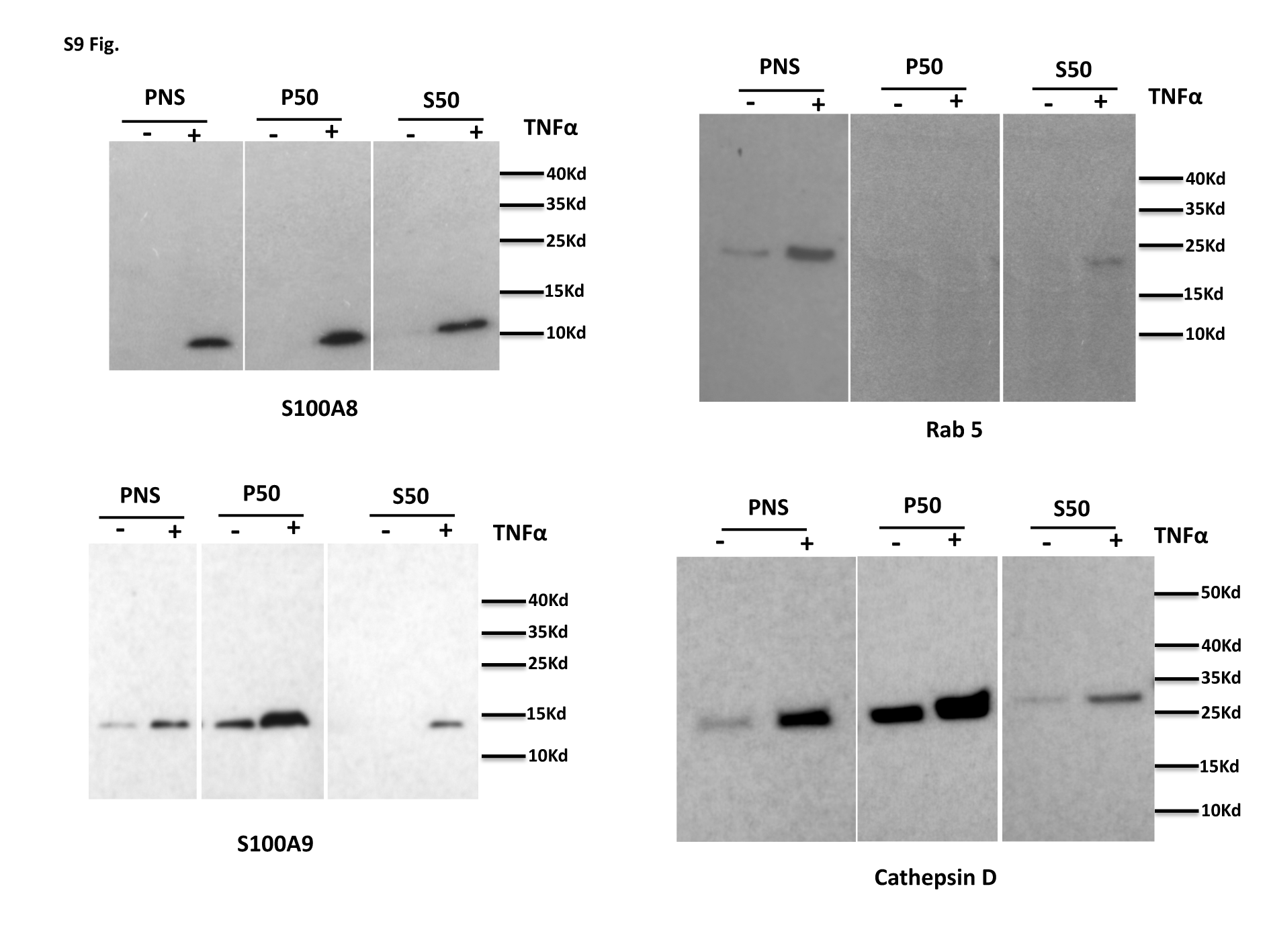

Supplement: S9 Fig — (TIF) [file pone.0145217.s009.tif]

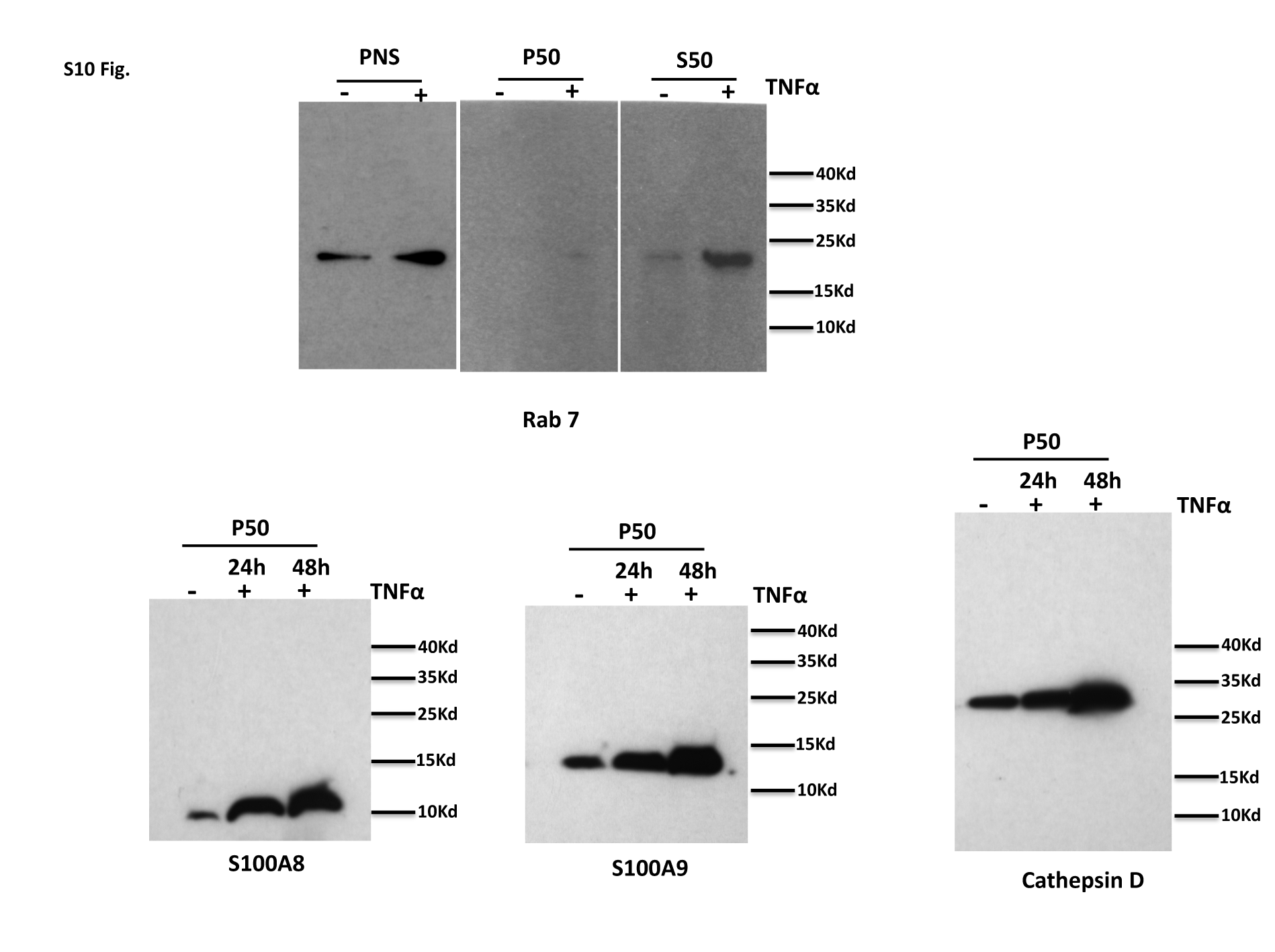

Supplement: S10 Fig — (TIF) [file pone.0145217.s010.tif]

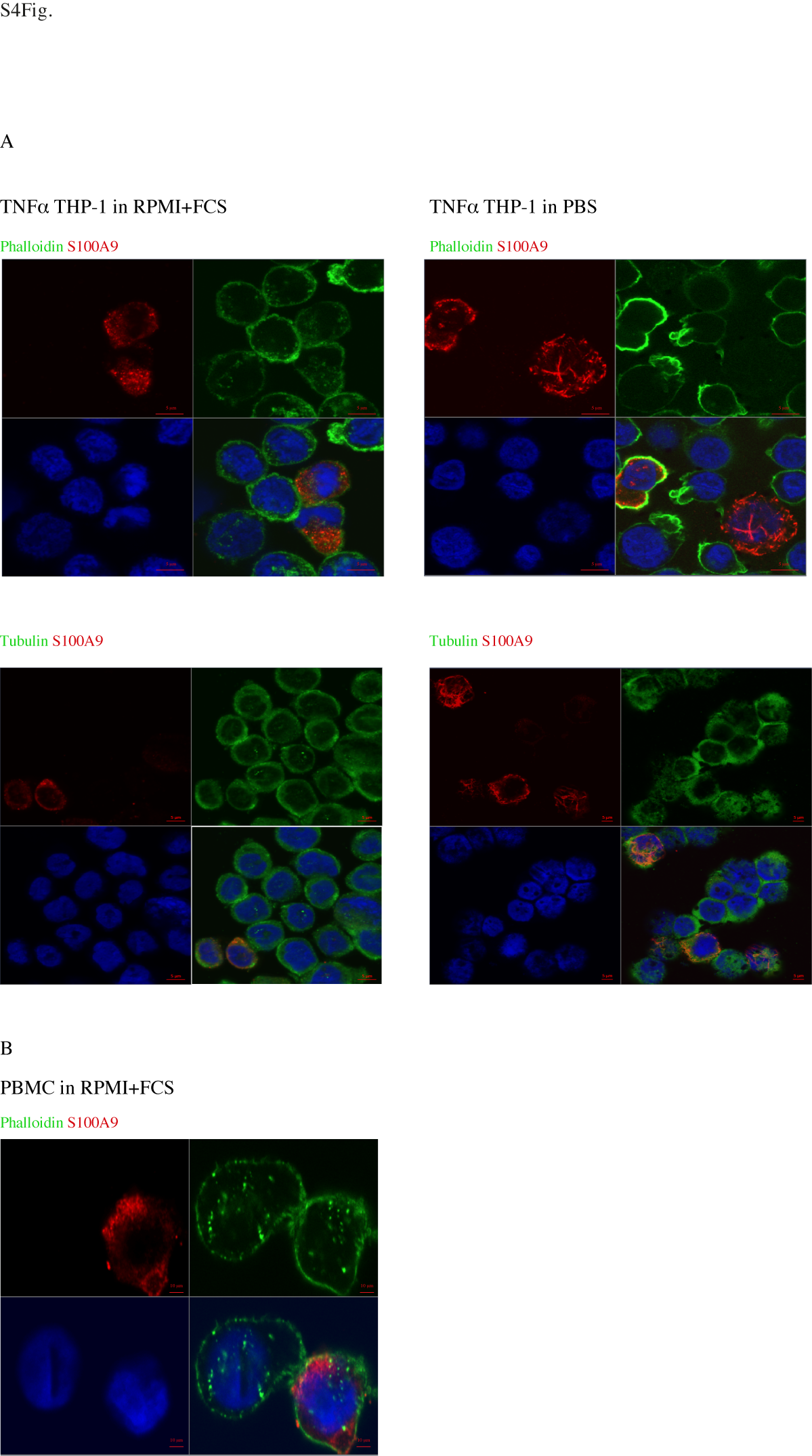

Supplement: S11 Fig — (TIF) [file pone.0145217.s011.tif]
